# Implementing SMS reminders for routine immunization in Northern Nigeria: a qualitative evaluation using the RE-AIM framework

**DOI:** 10.1186/s12889-022-14822-1

**Published:** 2022-12-17

**Authors:** Chisom Obi-Jeff, Cristina Garcia, Funmi Adewumi, Tobi Bamiduro, Winnie David, Alain Labrique, Chizoba Wonodi

**Affiliations:** 1 Department of Research, Direct Consulting and Logistics Limited, Federal Capital Territory, Abuja, Nigeria; 2 Department of International Health, International Vaccine Access Center, Johns Hopkins Bloomberg School of Public Health Baltimore, Baltimore, MD USA; 3 Department of International Health and Department of Epidemiology, Johns Hopkins Bloomberg School of Public Health Baltimore, Baltimore, MD USA

**Keywords:** Vaccination, Immunization, SMS reminder, Text message, mHealth, Qualitative study, RE-AIM, Nigeria

## Abstract

**Background:**

Short Message Service (SMS) reminders have improved vaccine uptake in low- and middle-income countries (LMICs). However, the limited use of SMS reminders in LMICs requires evaluating the intervention’s internal and external validity to improve adoption and sustainability. Using the Reach, Effectiveness, Adoption, Implementation, and Maintenance (RE-AIM) framework, we qualitatively assessed the impact of a SMS reminder intervention implemented in Kebbi State, Northwest Nigeria between May 20, 2019 and May 31, 2020. This will guide and inform future SMS reminder interventions to improve childhood immunization uptake in LMICs.

**Methods:**

In June 2020, we conducted 14 focus group discussions, 13 in-depth interviews, and 20 key informant interviews among 144 purposively selected participants from five local government areas of Kebbi State. For analysis, we used a deductive approach to develop preliminary codes based on the RE-AIM framework and the inductive approach to generate themes that emerged from the interviews.

**Results:**

The perceived importance and impact of the SMS reminder in improving demand and uptake for vaccinations were the consistent contributing factors that encouraged participants’ participation. Other facilitators included the involvement of health workers in supporting SMS reminder registration and community gatekeepers using existing structures to convey messages on scheduled immunization services. Policymakers adopted the intervention because it aligns with the state’s priority to improve immunization coverage. Similarly, the SMS reminder appealed to health workers and program managers because it reduced their workload and served as a performance monitoring tool to track immunization and intervention defaulters. Despite these, low mobile phone ownership and the inability to read text messages due to the low literacy level were the main barriers during implementation. Finally, data availability on cost-effectiveness and the intervention’s impact on improving coverage was critical for scalability.

**Conclusions:**

Our study demonstrated that SMS reminders in local languages could improve vaccination demand and uptake in resource-constrained settings due to their perceived importance and impact. Addressing the cited implementation barriers and promoting the facilitators is critical to its adoption and sustainability. Costing and impact data are needed to collaborate findings on the effectiveness of the SMS reminder to improve childhood vaccination uptake.

**Supplementary Information:**

The online version contains supplementary material available at 10.1186/s12889-022-14822-1.

## Background

Immunization remains one of the most cost-effective interventions available to public health today [1]. Globally, vaccines avert 2–3 million deaths yearly and prevent morbidity and disability from common and rare diseases [2]. Beyond saving lives and averting diseases, the benefit of vaccination extends across the life course of a vaccinated person [3]. For instance, vaccinations 1) prevent poor outcomes in the broader community by protecting the unimmunized population through herd protection, 2) stabilize health systems by providing opportunities for better primary healthcare (PHC) services, and 3) promote health equity by giving every child an equal chance and access to vaccination irrespective of their socio-economic status [3]. Furthermore, vaccination offers significant savings by avoiding the health costs associated with treating diseases and possible long-term disability and benefits national economies. For low- and middle-income countries (LMICs), it is estimated that every US$1 invested in childhood vaccination yields between US$19.8 and US$52.2 in return on investment from 2021 to 2030 [4]. However, countries attain these benefits when children receive all the recommended doses, which is crucial to achieving universal health coverage and the Sustainable Development Goals.

Despite introducing new and under-used vaccines, an estimated 20 million children globally did not receive the recommended three doses of diphtheria, tetanus, and pertussis vaccine in 2019 [5]. In the same year, ten countries accounted for 12.2 million unprotected children worldwide, with Nigeria reporting 3 million unvaccinated children [5]. In Nigeria, not all children benefit from immunization, as vaccine-preventable diseases account for an estimated 29% of childhood deaths annually [6]. While Penta3 coverage increased from 38% in 2013 to 50% in 2018 [7], the current immunization coverage rate falls short of national and global targets [5]. In 2018, about 31% of children received all vaccines by 23 months, and 19% had never received any vaccine from the RI program [7]. Furthermore, coverage is highly variable across the country, with the northwest and northeast regions reporting the worst performance [7].

Demand-related issues are reasons for non-vaccination in Nigeria, including lack of awareness, mother’s literacy level, weak mobilization, and vaccine hesitancy [8, 9]. A national survey revealed that lack of knowledge of the benefits of immunization and the place or time of vaccination accounted for 42% of the reasons why children remain unimmunized [10]. In Northern Nigeria, lack of awareness was the consistent reason for the non-vaccination of children aged 12–23 months in North Central (50%) [11], North East (39%) [12], and North West (37%) [13]. Similarly, a study in Northern Nigeria found that lack of knowledge about vaccines and vaccination services (50%), mother forgetting or being too busy (16%), and suboptimal access to RI services (15%) were the commonly reported reasons for non-vaccination [14]. Hence, an effective and innovative strategy is needed to enhance vaccine demand and uptake, overcome these information and knowledge gaps, and improve compliance with the immunization schedule.

Evidence has shown that reminder and recall systems improve health-seeking behavior in vaccination [15]. A Cochrane review found that patient reminder or recall interventions in PHC settings increased the number of people who received vaccinations, whether sent when vaccinations were due or overdue [16]. While postcards, text messages, and automated telephone calls were efficient, telephone reminders were more effective but costly than other methods [16]. Thus, there is a need for efficient and cost-effective text message interventions [16]. Mobile phone text messaging, also known as the short messaging system (SMS), is a cost-effective method of disseminating health information and reminders, especially among low-income populations [17, 18]. In addition, mobile phones, including non-smartphones, are becoming widespread in LMICs given their low-cost nature [19], thus making them a convenient tool to improve health outcomes [19, 20]. In Nigeria, there is an opportunity for mHealth to support child health programs to improve health outcomes [21], as mobile phone ownership, availability (i.e., access), and usage (i.e., penetration) is considerably high at 63.3%, 64%, and 64%, respectively [22]. Nevertheless, there is a significant mobile phone ownership gap by location and gender. Mobile phone ownership in urban areas is high at 77.8% compared to rural areas at 53.6%, a 31.4% gap [22]. Similarly, the mobile phone gender gap stands at 13.1%, with 69.7% of males owning a mobile phone compared to 56.7% of females [22]. Among those who do not own a phone, the most reported reasons were that they could not afford one (56%), no electricity to charge the phone (26%), and no mobile coverage signal where they live (20%) [22].

SMS reminders have successfully improved immunization uptake in LMICs in small-scale clinical and community settings [23–27]. While this is promising and has strengthened immunization programs in developing countries, its effectiveness varies with the implementation context [28]. Furthermore, the limited attention and use of SMS interventions in LMICs requires evaluating the intervention’s internal and external validity to improve sustainable adoption.

Studies have emphasized the extensive use of dissemination and implementation models, theories, and frameworks to understand, guide, and inform future interventions for the scale-up [29]. Several frameworks to determine implementation success for translation into practice exist. One such framework is the Reach, Effectiveness, Adoption, Implementation, and Maintenance (RE-AIM) framework used in several studies to design, implement, and evaluate interventions “with a higher likelihood for uptake and sustainability in a typical community or clinical setting.” [30, 31] The five RE-AIM dimensions are defined in Table 1.

**Table 1:** Definition of RE-AIM dimensions

Dimension	Definition
Reach	The number, proportion of the intended audience, and participants’ representativeness compared with the intended audience
Effectiveness	The degree to which the intervention changes health outcomes and quality of life, including producing unintended or negative results
Adoption	The number and proportion of settings and staff members who agree to initiate program or policy change and how representative they are of the intended audience regarding the location and the staff
Implementation	The degree to which those settings and staff members deliver a program or apply policy as intended, including the adaptations made and the related costs
Maintenance	The sustained effectiveness at the participant level and sustained (or adapted) delivery at the setting or staff level

Several authors have recommended using qualitative and quantitative approaches to help understand each RE-AIM dimension [31, 32]. Unfortunately, most RE-AIM studies assess intervention outcomes quantitatively and lack qualitative contribution. Additionally, there is limited use of qualitative methods with RE-AIM [33, 34], thus, limiting the ability to understand why and how outcomes were obtained.

To the authors’ best knowledge, there is no evidence of using the RE-AIM framework to assess SMS reminder interventions in LMICs. For this study using the RE-AIM framework, we qualitatively evaluated the implementation of a SMS reminder intervention called the Immunization Reminder and Information SMS System (IRISS) in Kebbi State, Northwest Nigeria. This is to guide and inform the implementation of SMS reminder interventions to improve childhood immunization uptake in developing countries.

## Intervention

IRISS, also known as *Tunatar da ni* (meaning “Remind me” in the Hausa language), is a cloud-hosted customized registration and message scheduling application that provides automated registration and delivers one-way immunization information to the targeted population via SMS. The application houses four databases: Contact information, Child data, Health facility information, and a Message library detailed elsewhere [35]. IRISS used SMS to inform and educate the public about the importance of immunization and remind caregivers/parents of their child’s immunization schedules, including the vaccination schedules of health facilities in their locality. We designed the project as a two-arm cluster randomized controlled trial to assess the IRISS intervention’s impact on improving demand for and uptake of immunization. Fourteen (14) Local Government Areas (LGAs) received the SMS intervention for one year between May 20, 2019, and May 31, 2020, while the remaining seven (7) LGAs did not receive the intervention.

IRISS delivered immunization information to the public and parents/caregivers in the 14 intervention LGAs in three ways:

1. *General broadcasts*
  IRISS sent general broadcasts to 190,000 active subscribers every quarter to improve positive norms about vaccination. The broadcasts contained informative messages about immunization, prompting them to opt-in to the IRISS e-registry for more information. Individuals that responded to general broadcasts were called Leads and scheduled for the weekly targeted broadcasts.
2. *Targeted broadcasts*
  IRISS sent two targeted broadcasts every week. Every Thursday, IRISS sent messages on immunization benefits to 3,924 Leads, 1,010 community gatekeepers (District Heads, Village Heads, *Mai-ungwas*, Imams), and 406 health workers. Every Sunday, immunization days for the nearest health center are sent to 1,010 community gatekeepers to pass on to their communities and congregations using existing structures. Within the intervention period, IRISS sent 160,428 and 15,150 targeted broadcasts on the benefits of immunization and health facility schedules, respectively.
3. *Individualized/personalized reminders*
  21,906 vaccine-age children (approximately 61% of the targeted population) were registered into the IRISS application. During the intervention period, IRISS sent 36,722 individualized reminders to parents/caregivers a day before their child’s immunization due dates.

Registration to receive messages from IRISS was via direct or proxy. Direct registration was for caregivers or community gatekeepers who own a phone. In contrast, proxy registration was for those who did not have a phone but could access a relative/friend’s phone to receive messages.

IRISS disseminated all messages in the local Hausa language. Table 2 provides example SMS messages for general and targeted broadcasts and personalized reminders.

**Table 2:** Sample IRISS intervention messages by broadcast and language type

Broadcast type	Sample message in English	Sample message in Hausa
General broadcast	Immunize your child with a single dose of the BCG vaccine at birth to prevent tuberculosis. To learn more, text YES to 07040055505	Ka/ki ba wa yaron ka/ki rigakafin BCG da zarar an haife shi/ta domin kariya daga tarin huka. Domin karin bayani game da rigakafi, tura yes ga 07040055505
Targeted broadcast to Leads, community gatekeepers, and health workers	Promoting the health benefits of vaccines is a collective effort. Do your part by telling relatives, neighbors, and friends to vaccinate their children	Sanar da jama'ar mu amfanin kiyon lafiya abu ne da ya rataya a wuyan kowa. Ka bayar da taka gudunmawa ta hanyar sanar da yan'uwa da sauran alumma domin su kaia yaransu wurin rigakafi
Targeted broadcast to community gatekeepers	Greetings! PHC Biri will be conducting an immunization session on Monday. Please inform your community members	Barka! Akwai allurar rigakafin yara a ranar Litini a PHC Biri. Taimaka ka yada wanan sako a cikin unguwar ka
Individualized/ personalized reminders	Greetings! Yusuff is due for Measles vaccination tomorrow; kindly visit Illela PHC. They conduct immunization sessions on Thursday and Monday	Barka da warhaka! Yusuf ya isa karbar rigakafin Measles gobe, ka garzaya zuwa Illela PHC. Su na rigakafi a ranar Alhamis,Litini

## Methods

### Study design

We used Focus Group Discussions (FGDs), In-depth Interviews (IDIs), and Key Informant Interviews (KIIs) to evaluate the impact of the IRISS intervention on all RE-AIM dimensions at the end of the intervention. For each RE-AIM element, we adapted qualitative questions from Glasgow et al. [30] and Holtrop et al. [32] to understand how and why outcomes occurred, their variation across settings, and how implementation context may influence generalizability and translation to other locations. Table 3 shows the adapted qualitative questions for each RE-AIM dimension evaluated. We used the Standards for Reporting Implementation Studies [36] (Additional File 1) and the Standards for Reporting Qualitative Research [37] checklists as a guide in reporting this study.

**Table 3:** Applied qualitative questions for each RE-AIM dimension

Dimension	Qualitative questions
Reach	• What type of IRISS messages did people receive? • What are the facilitators and barriers to registering into IRISS for community members? • What are the reasons for not registering for IRISS across study sites? • What was done to encourage participation?
Effectiveness	• Did the IRISS intervention improve uptake and demand for vaccination? • What other factors contributed to the outcomes observed? • How meaningful were the outcomes for different participants?
Adoption	• What factors contributed to the State Primary Health Care Development Agency (SPHCDA) and its staff, including health workers and program managers, participating in the IRISS intervention? • What were the reasons for participating in the IRISS intervention or not? • What were the barriers to successful adoption across health workers and their settings? • Was there a partial or complete adoption?
Implementation	• How was the IRISS intervention implemented? • What influenced implementation or the lack of it? • What were the modifications to the intervention, and why did they occur? • What were the contextual factors and processes underlying barriers at each setting to registering participants into IRISS, and how was it addressed? • How did health workers improve registration?
Maintenance	• Was the IRISS intervention sustained, discontinued, or modified- and why? • What are the barriers to maintaining the intervention? • Is the IRISS intervention being implemented (and adapted) after the intervention core period?

### Study setting

We conducted our study in Kebbi State, Northwest Nigeria, because of its low RI coverage due largely to a lack of vaccination awareness [10, 13]. Kebbi State has a population of about 4.2 million, of which 99% are Muslim [38]. It is primarily rural and comprises 21 local government areas (LGAs) divided into four Emirate Councils – Argungu, Yauri, Zuru, and Gwandu –and 225 political wards. The main economic activity is agriculture, and the primary language is Hausa.

According to a recent survey, 36% of the households in Kebbi State are in the poorest quintile, and only 2% are in the wealthiest [7]. Among individuals aged 15–49 years, only 15% of women and 35% of men were literate^1^ [7]. Likewise, 22% of women and 63% of men own a mobile phone [7]. In addition, immunization services are delivered via government hospitals, PHCs, and mobile/outreach stations. While 6% of infants 12-23 months received all basic vaccinations,^2^ a whopping 21% have never been vaccinated [7]. Furthermore, RI coverage in Kebbi State is low at 11% compared to 50% nationally [7].

In Kebbi State, the Study Advisory Group and the study team selected five LGAs for the qualitative study – Aliero, Argungu, Fakai, Shanga, and Augie – based on the following criteria (see Table 4):

1. Representation from the four Emirate Districts and study intervention and control LGAs
2. Demography: Inclusion of rural and urban areas
3. RI performance [39]
4. Phone ownership [39]

**Table 4:** Characteristics of study LGAs

S/N	Name of LGA	Emirate Council	Study Group	Demography	% RI performance^a^	% Phone ownership^b^
1	Aleiro	Gwandu	Intervention	Rural	23%	45%
2	Argungu	Argungu	Control	Urban	10%	42%
3	Fakai	Zuru	Intervention	Rural	35%	5%
4	Shanga	Yauri	Control	Rural	53%	3%
5	Augie	Argungu	Intervention	Rural	20%	2%

^a^RI performance is defined as the proportion of children appropriately immunized for age assessed from the Q3 2018 Lots Quality Assurance Sampling survey. High performance > 16% and Low performance < 15%

^b^Phone ownership is defined as owning a phone or having access to a friend, relative, or neighbor who owns a mobile phone

### Study participants and sampling

We purposively selected participants for the interviews from the five LGAs. They included RI program managers (i.e., state-level policymakers, program managers at state and LGA levels, and development partners) and PHC health workers with a minimum of one year of experience in their current positions. These participants were identified and recruited for the qualitative interviews through state members of the Study Advisory Group, the Kebbi State Primary Health Care Development Agency, the Kebbi State Emergency Routine Immunization Coordinating Center, and the LGA program managers based on prior knowledge of their characteristics and eligibility status. We contacted the identified RI program manager and PHC health worker by email or phone to inform them of the study and confirm their interest and availability to participate in the interviews. 

Also selected for the interviews were the community-level participants who had been current community residents for at least one year. They included parents of newborns and children under five years of age, pregnant women, opinion leaders (traditional and religious leaders, persons of influence), ward development committee (WDC) members, traditional birth attendants, young men and women, and male and female youth groups. We identified the participants for the interviews at the community level through their community leaders (District Heads and *Mai-unguwas*) and local RI provider at the PHC. We approached these individuals to inform them about the study and confirm their interest and availability to participate in the interview.

For all the participants, we also followed up with a phone call for recruitment at least one day before the interview to reconfirm their interest and availability to participate in the scheduled interview. Invited participants were not required to have a phone or have experience using a phone. All participants were available and consented to participate in the discussions.

Before the interview, the data collectors obtained consent in person using a standard oral consent script. The interviewer read the verbal consent script to the potential participants and answered any questions they may have had. The consent was administered in English, the official language widely spoken in Nigeria, or Hausa where necessary.

### Data collection

We developed the data collection instruments and conducted 14 FGDs, 13 IDIs, and 20 KIIs among 144 people in the study LGAs between June 22 and 27, 2020. Additional Materials 2 and 3 present the interview guides and a complete list of respondents and interview types. After obtaining verbal consent, trained research assistants and data collectors conducted the interviews using a pre-tested, semi-structured interview guide at the participant’s home or a convenient location. We conducted 27 interviews at the community level in the Hausa language due to the literacy level of the study setting. We also collected participants’ socio-demographic information^3^ using a cover sheet. Each interview lasted between 60 and 90 min. The study team offered no compensation to participants during the data collection. However, we provided FGD participants 500Naira in travel per diems.

While the data collection happened during the COVID-19 pandemic, data collection activities were not affected. During the data collection period, there were no lockdown or movement restrictions in the study sites, and no specific restrictions on research activities were issued by the National Health Research Ethics Committee of Nigeria (NHREC). For our in-person data collection activities, we adopted the approach and guidelines of the National Primary Health Care Development Agency (NPHCDA) and the Nigerian Center for Disease Control (NCDC) for face-to-face interaction. Adapted from the World Health Organization [40], the NPHCDA guidelines supported the safe continuation of face-to-face service delivery, provided modifications to services provisions were made, including face masks, social distancing, crowd avoidance, and hand and respiratory hygiene [41, 42]. In addition, NCDC provided protocols for in-person activities for businesses and employers, requiring all individuals to observe the recommended non-pharmaceutical interventions in public places to prevent exposure to coronavirus transmission [43]. Our in-state study coordinator and data collectors followed all COVID-19 preventive guidelines, such as temperature testing using an infrared thermometer, handwashing with soap and water before commencing interviews, and maintaining 2 m distance between each other before and during the interviews. We successfully conducted the face-to-face qualitative interviews with no suspected COVID-19 cases among the field staff and the study participants.

### Data analysis

The audio-recorded interviews were transcribed verbatim and analyzed using a deductive and inductive approach. We engaged external vendors to transcribe all interviews. Interviews conducted in Hausa were transcribed and back-translated to English for analysis.

We used deductive and inductive methods for the qualitative data analysis [44]. We developed a preliminary codebook with the initial set of codes based on the RE-AIM framework (deductive approach) to guide the analysis. This codebook defined all codes with rules for application. Then, ten transcripts were randomly selected from the study sites and participants and read independently by COJ and FA for content comprehension and to generate new codes emerging from the data (inductive approach). COJ, FA, and CW reviewed and compared the codes for harmonization and finalization in the codebook.

**Fig. 1:**
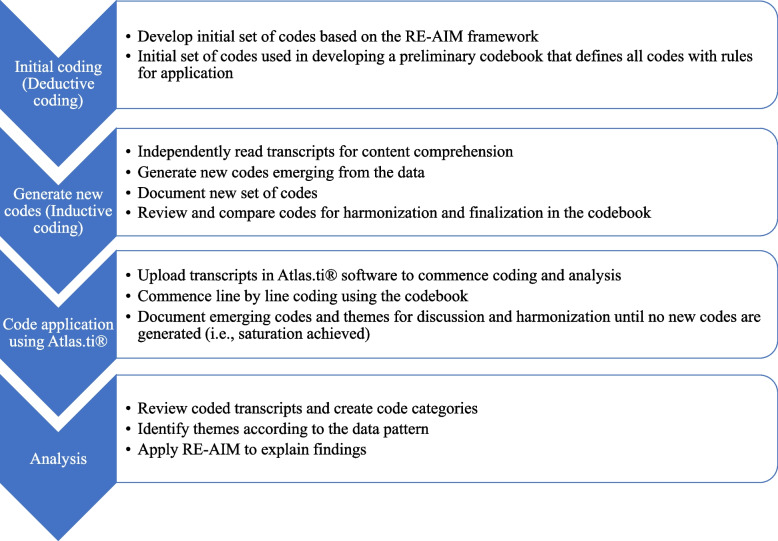
Overview of the deductive and inductive analysis process

COJ entered the transcripts in Atlas.ti® software and coded each transcript line by line using the codebook. Upon reading and coding subsequent transcripts, emerging codes and themes were recorded and shared with the team for discussion and harmonization. As a result, the codebook was revised and modified in an iterative process until no new codes were generated. The study team further reviewed the harmonized codes to form relevant code categories and appropriate themes according to the data pattern for analysis. This review ensured that the codes within the themes are meaningfully connected while presenting a clear distinction between the themes. The data was queried for meaningful content using Atlas.ti® and interpreted based on identified themes captured with the evaluation (i.e., RE-AIM) questions. The themes were analyzed by respondent type, study group (intervention and control LGAs), and setting. Figure 1 presents an overview of the deductive and inductive analysis process. The respondents’ socio-demographic information was analyzed in Microsoft Excel and submitted as frequencies and percentages.

## Results

### Study participant’s demographic characteristics

We interviewed 144 participants representing the target population at all levels. Most were community members, 124 (86%), and 99 (69%) were males. Their ages ranged from 19 to 77 years with an average mean of 39 years (standard deviation ± 13.3). Educational status among our participants was slightly high. More than half of our participants, 113(78%), had a formal education, and about 72% attained secondary school or higher (Table 5).

**Table 5:** Socio-demographic characteristics of participants

Characteristics	Number (*n* = 144)	Percentage (%)
***Respondent type***		
Community members	124	86%
Government program managers	9	6%
Government health workers	10	7%
Policymaker	1	1%
***Sex***		
Male	99	69%
Female	45	31%
***Attended formal education***		
Yes	113	78%
No	31	22%
***Highest level of formal education completed***		
None	31	22%
Primary School	9	6%
Secondary School	39	27%
Graduate	62	43%
Post-Graduate	3	2%

### Reach

For the Reach dimension, we assessed the receipt of IRISS messages, facilitators, and barriers to registering into IRISS among community members and activities conducted to increase participation.

Most participants from the intervention LGAs confirmed to have received IRISS messages such as 1) a reminder of their child’s vaccine due dates on their mobile phones or via proxy, 2) a message on the immunization schedule of the nearest facility, 3) a message on the importance of vaccination, and 4) a message on COVID-19.

“***Participant 1:**** Sometimes it’s a reminder to take the children to the hospital. Some other times its awareness about immunization. ****Participant 2:**** They also tell us to take these persons to the hospital on these dates for immunization; that’s why they send it. But some days back, we were sent messages on coronavirus. ****Participant 3:**** Honestly, I did not receive them because the messages come through my wife’s phone to take the child.”-* Opinion leaders.

The key facilitators of involvement in the IRISS intervention were its perceived importance in reminding and educating community members on the importance of immunization and satisfaction with the message content, especially the health facility day and operation time accuracy.

“***Participant 1:**** It is very good because if you have your phone and you get the message, it would remind you to go for immunization. ****Participant 2:**** It is very useful where you do not intend on taking the child, or you have forgotten; it’s a reminder even if it’s your husband that will be reminded.”* – Mothers of children under-five.

Most participants cited the lack of mobile phones and the inability to read text messages as barriers to non-participation in IRISS. However, educated community members were perceived not to encounter such challenges.

“***Participant 1:**** Yes, most of them are not educated. Some do not have a phone or even never bought one. If the message is sent to him, it is of no use to him. ****Participant 2:**** Tunatar da ni has its disadvantage and advantage. The disadvantage is that we cannot read. We find it difficult to read the message and other villagers also have this problem*.” - Fathers of children under-five.

However, most mobile phone owners with a low literacy level sought help from relatives or other people who could read it.

Despite the low phone ownership and literacy levels, involving trusted stakeholders in the community, such as the health workers who served as proxies for receiving the SMS for both community gatekeepers and parents, encouraged participation.

*“The major challenge we face is that most people do not have phones…You will have to register for most people here because they don’t have phones, both mothers and fathers.”* - Health worker.

Similarly, disseminating weekly performance reports to program managers at State and LGA levels for follow-up and the occasional supportive supervision where the study team and state program managers conducted on-the-job training for health workers encouraged registrations into IRISS.

### Effectiveness

The effectiveness dimension assessed the perceived impact of IRISS in improving demand and uptake for vaccination and its usefulness for different stakeholders.

Many participants noted that personalized reminders reminded caregivers, especially those who were busy and forgot their child’s vaccination dates and prompted them to go for vaccination.

“*Before, we were not bothered with immunization. But now, there is a constant reminder of when and where to be immunized. A woman in my compound would come to meet me and show me a text message, and she would ask me to read the content of the text message for her. I will read the message telling her it is time to immunize her child. She will then take her child for immunization*.” - Female Youth.

The alluded perceived impact of the IRISS intervention in improving demand for vaccination and immunization coverage in the state could have implications for scale-up and sustainability.

“*IVAC too, has come in to do the intervention where women and caregivers are reminded about routine immunization of their children using SMS. This impacted positively in creating demand for immunization which I believe has also contributed towards improving coverage for immunization.*” –Policymaker.

Receiving IRISS messages also increased awareness and knowledge about vaccination and triggered discussions about immunization in the community.

“*I was not aware that those diseases could be cured, but because of the Tunater da ni message, I later knew.”* - Father of children under-five.

The IRISS intervention helped reduce the workload of health workers and program managers from following up on clients on the child’s subsequent immunization due date.

“*It has greatly reduced the stress I used to have. Those receiving the texts have been turning up promptly.*” –Health worker.

Informed of how the IRISS intervention works, program managers and health workers from the control LGAs also felt it would ease their workload and improve health outcomes.

“*Since it’s easier for them to know when it’s due time, and there won’t be any need to make an announcement, you will just receive a message reminding you to come for immunization*.” –Health worker.

Program managers at state and lower levels also mentioned that IRISS messages helped them monitor health workers’ performance in conducting registrations as instructed and tracking immunization defaulters as proxies.

“*As you know, from time to time, I receive a message from Tunatar da ni about the number of people registered and the number of hospitals in our local government. As soon as I receive the message, I call the RI providers and praise those who do well and those behind.”* - Program Manager.

### Adoption

For the RE-AIM adoption dimension, we assessed the facilitators and barriers to adopting the IRISS intervention among the state institutions – State Primary Health Care Development Agency (SPHCDA) and implementing staff (health workers and program managers). State decision-makers adopted the IRISS intervention because improving immunization coverage is a priority in Kebbi State, and they welcomed interventions to achieve this goal. The IRISS intervention was included as part of the activities assessed during the state’s RI supportive supervision at the health facilities.

Implementing staff of the agency and partners were committed to improving RI coverage in the state and providing support. For example, state program managers supported supportive supervision at health facilities to encourage registration, and health workers conducted registrations, sensitized community members about IRISS, and helped caregivers understand the messages.

“*Whenever I access a parent, I try to collect their phone number and ask them to be attentive to their phone because a message will be sent when next to bring their kids for another vaccine. Sometimes the parents come to me for an explanation whenever a message is sent to them*.”- Health worker.

Among the 426 health facilities providing RI services in the intervention LGAs, 361(85%) participated in conducting registrations. Health workers from the remaining 65 health facilities reported network issues and a lack of mobile phones as barriers to successful adoption.

Although the IRISS intervention was implemented in 14 LGAs as a research project to inform policy actions, decision-makers expressed willingness to scale up to the remaining seven (7) LGAs given the perceived impact.

“*We are thinking of scaling up …If you compare what is happening in these 14 LGAs, we will likely see a positive impact, so scaling up is truly something we want to do*.” –Policymaker.

### Implementation

The implementation dimension explored the intervention fidelity, including motivations for/barriers to some aspects of the intervention. The most frequently cited implementation barriers were low mobile phone ownership, especially among rural dwellers, and the inability to read text messages due to low literacy. This may have more implications on the impact of the intervention than initially planned, especially for the uneducated.

We also learned from the participants interviewed that most community members who own mobile phones use them to receive calls only and ignore text messages because they either cannot read, are unaware when they receive these messages, or avoid paying attention due to unsolicited messages.

“*That’s true. Most people only use the phone to receive and make calls and are not even bothered when messages come in.”* –WDC member.

Despite these challenges, those who opted to receive SMS via proxy noted that their proxies (e.g., health workers, relatives, or friends) informed them when messages were received.

“*He knows the importance because he tells me about it whenever he receives the same*.” –Mother of a newborn.

When informed by the health workers, the community gatekeepers used town announcers to convey messages on scheduled immunization services in the nearest health facility.

“*When the time comes for immunization, the Mai-anguwa* [Village Head/Chief] *is informed. He will, in turn, inform the announcers to pass the information across the town..*” - Opinion leader.

Beyond the commitment to the state target and avoiding sanctions, health workers’ willingness to serve as proxies was driven by the desire to improve health outcomes.

“*The reason for carrying out this decision is that immunization is important. I endeavor to provide enlightenment, so they know that vaccination benefits their children’s wellbeing*.” –Health worker.

While health workers mentioned that they informed parents and community leaders registered on their phones, only one was clear on how she did it.

“*I follow them up, house-to-house, to get the message across to them.*”- Health worker.

Meanwhile, implementing the door-to-door/house-to-house vaccination by the SPHCDA provided another opportunity for health workers to register eligible children and sensitize their parents. Our data showed that most registrations by health workers were done during an outreach session.

To further encourage registrations during the intervention delivery, the study team provided a modest incentive (6 Naira) per successful registration to encourage RI providers to conduct proxy registrations. To promote and improve registrations, state program managers and the study staff led supportive supervision in health facilities with inconsistent registrations and a high target population. Health workers were encouraged to inform community gatekeepers and other health workers when they received their messages.

While State program managers encouraged proxy registration using the phone numbers of friends and relatives, they felt using a health worker was a limitation in the intervention implementation. They felt that health workers might not convey the information to the recipients.

State program managers also felt that incorporating Robocalls as part of IRISS intervention for the uneducated would have been more effective due to the low literacy level in the study settings.

“*Robocall, ahh… it will go along in disseminating the positive information because you can send a text message and they cannot read. But in those areas that can read, they can be sent SMS*.” –Program manager.

Community members agreed to have the educated ones receive the SMS reminder, and the uneducated get the messages via phone calls or town announcers.

“**Participant 2**: *Only educated people should be registered for SMS, while the uneducated ones should be given a reminder call*. **Participant 3:***Those with phone and telecommunication services should be registered. For those without, a town crier should be provided to them*.” - Fathers of children under-five.

### Maintenance

We assessed the IRISS intervention sustainability and the reasons for continuation or discontinuation to inform future program design and scale-up. State decision-makers noted that support for the intervention’s sustainability depends on the available data to show its impact on improving coverage and affordability by the state government.

“*Yeah... uh… it’s data because we leverage on using data for action*.” –Program Manager.

The data will be used for decision-making by key stakeholders who would prioritize and approve its inclusion in the state budget for funding. Upon funding, the IRISS intervention would be situated and managed by the immunization department of the Kebbi SPHCDA.

Although we discontinued the SMS-reminder dissemination on immunization after the project ended in May 2020, the NCDC used the IRISS application to send messages on COVID-19 preventive measures in the Hausa language across the 21 LGAs between June 5 and July 31, 2020. Most participants in both study groups received messages from IRISS on COVID-19.

## Discussion

We found considerable facilitators to optimize the reach, effectiveness, adoption, implementation, and maintenance of the IRISS intervention within community settings to improve the demand and uptake of immunization services. The perceived importance and impact of the IRISS intervention in enhancing the demand and uptake for vaccination were the main contributing factors that encouraged participants’ participation. The IRISS intervention served as a reminder, prompted vaccination visits, increased awareness and knowledge about vaccination, and triggered immunization discussions among community members. This is consistent with other studies where SMS reminders increased vaccination appointment attendance and uptake [45, 46]. Similarly, embedding information on vaccination benefits has improved influenza vaccine uptake among low-income, urban, and minority populations [47], made parents feel encouraged and aware, and helped them influence attitudes toward self-responsibility [48]. While our study did not investigate the particular message that substantially impacted parental behavior, messages informing and educating the public about the importance of immunization are needed to help community members move from lack of awareness to considering vaccination [49].

The community members’ desire to participate in the IRISS intervention due to its perceived impact aligns with the state decision-makers and service providers. State decision-makers adopted the IRISS intervention because it aligns with the policy priority to improve immunization coverage. Interventions that address the target population’s health needs have been shown to promote acceptability and political will to devote resources to implementing and scaling up the program in LMICs [50]. This is particularly important in areas with competing health priorities and limited resources to ensure the intervention’s sustainability after project funding ends. While our study found that the staff and partners attached to Kebbi SPHCDA supported the implementation of the IRISS intervention and tracked registrations during supportive supervision, sustainability depends on the availability of impact and cost-effectiveness data. This is similar to other studies in LMICs, where the availability of research and monitoring and evaluation data facilitated the scale-up of public health interventions [50].

Additionally, despite the desire to improve RI coverage in the state, the IRISS intervention appealed to health workers and program managers because they believed it reduced their workload and served as a performance monitoring tool to track immunization and project defaulters. Previous studies reported that SMS technology helped service providers monitor and provide accurate information on patients’ acne treatment adherence [51] and improved case-management practices [52]. It may be worth considering sending SMS reminders to service providers and patients as an essential addition to immunization reminders to improve health outcomes.

Other factors that encouraged participation at the community level were health workers’ involvement in supporting IRISS registration and sensitization. These individuals are trusted stakeholders who influenced the acceptability of a vaccination reminder intervention in Kebbi State [53]. Their participation in delivering the intervention to encourage community participation cannot be overemphasized. Our health workers did not report that incentives encouraged support to register community members. However, adding incentives for involvement in SMS reminder interventions has promoted participation [27, 54], but there may be cost implications in sustaining this approach in LMICs. As implementation progressed, program managers’ refresher training during occasional supportive supervision reinforced the initial training received by health workers and filled the gap for new health workers to encourage more registrations. The weekly performance SMS messages to program managers also served as an accountability measure to monitor different health workers’ participation. In addition, satisfaction with the accuracy of the health facility schedule sent directly to caregivers’ mobile phones encouraged participation in our study. This is similar to another study where a high level of satisfaction with SMS reminders’ precision was helpful and motivated parents to recommend the text messages to other parents [47].

Across the RE-AIM framework, we found that the low mobile phone ownership in rural settings and the inability to read text messages due to the low literacy level were the main barriers to non-participation in IRISS. The baseline assessment also reported this as a potential barrier to implementing the IRISS intervention [35]. These findings are similar to the findings of other studies in LMICs that cited these same barriers to SMS reminder intervention implementation [55–58]. This might directly impact the efficacy of SMS messages in improving immunization coverage. While the IRISS application disseminated the SMS messages in Hausa, this study reveals that low literacy could be a determinant of not owning a mobile phone. In our research, people used the health worker, family member/relative, or friend’s phone to receive immunization messages and reminders where phone ownership was lacking. Incorporating proxy registration should be considered for future SMS-reminder interventions for childhood vaccination appointment reminders for caregivers who do not own a phone. Also, using town announcers to disseminate information on vaccination dates, times, and venues could be an opportunity to reach the unreached. Another suggestion would be for communities to establish a microcredit program where community members can purchase a group phone via loan [59]. However, its feasibility and acceptability will need to be assessed.

The findings from this analysis and the baseline analysis indicate most community members who own mobile phones use them to receive calls only, ignore text messages, or do not know how to open SMS messages [35]. This is consistent with barriers encountered in other developing countries [58]. This limitation could be due to low literacy, exposure to mobile technologies, or unhappiness with unsolicited messages over time. In other studies, the reported drawbacks were people not reading their text messages and being annoyed at receiving multiple messages, but no SMS reminder has caused adverse iatrogenic results [46]. Incorporating phone calls or Robocalls in SMS reminder applications could be a simpler alternative for those who cannot read and do not know how to open text messages. Costing implications and feasibility need to be assessed in low-resource settings. The use of two-way messaging that elicits a response from message recipients to address the challenge of ignoring text messages has been proposed [46]. Further research is recommended to ascertain the feasibility of short-term interventions such as childhood vaccination appointments. This is because evidence has shown two-way messages to be more effective for life-long programs requiring counseling and communication with a health provider, such as HIV appointments [60].

Despite the perceived importance and impact of the IRSS intervention, addressing the cited implementation barriers and promoting the facilitators is critical to its sustainability. Likewise, data on the intervention’s effectiveness in improving immunization coverage and the cost-effectiveness for scalability is needed. 

## Limitation

Our study had several limitations. Given that the study methodology relied on a small sample size to explore an in-depth analysis of the IRISS intervention, our findings are based on a limited number of participants and are subject to social desirability bias, especially the effectiveness dimension. Thus, our study findings are unique to settings and populations with similar contexts and cannot be generalized. Despite these limitations, we explain results obtained across the RE-AIM framework and how we achieved these results, including implementation barriers and facilitators. We also present lessons to overcome future failures and considerations for scale-up or adaptation in other comparable contexts.

Additionally, the interview guide used for the assessment was generic and may not be appropriate for some participants, given the different contexts and cultures in Kebbi. Nevertheless, our analysis approach compensated for this limitation. We immersed ourselves in the data to find excerpts that fit the initial set of codes based on the RE-AIM framework but then inductively developed new codes from the data and iterated on the codes as we sifted through the data. This helped us understand how and why different patterns of results occurred for various outcomes, participants, and settings to influence translation to other locations.

## Conclusion

We conclude that the IRISS automated one-way reminders in local languages could improve vaccination demand and uptake in resource-constrained settings due to its perceived importance and impact. The barriers identified present learnings to inform future community-based SMS-intervention design and implementation to improve uptake and increase sustainability. In addition, costing and impact data are needed to corroborate findings on the effectiveness of the IRISS intervention to improve childhood vaccination uptake. Targeted interventions are required for the uneducated and those without access to mobile phones to benefit from the intervention.

## Supplementary Information

**Additional file 1.** Standards for Reporting Implementation Studies Checklist.

**Additional file 2.** Interview Guides.

**Additional file 3.** Complete list of participants interviewed and type of interviewconducted.

## Data Availability

The dataset generated or analyzed during this study is included in this published article and available from the corresponding author upon reasonable request.

## Abbreviations

FGD: Focus Group Discussion
IDI: In-depth Interview
IRISS: Immunization Reminder and Information SMS System
KII: Key Informant Interview
LGA: Local Government Area
LMIC: Low- and Middle-Income Countries
NCDC: Nigerian Center for Disease Control
NPHCDA: National Primary Health Care Development Agency
PHC: Primary Health Care
RE-AIM: Reach, Effectiveness, Adoption, Implementation, and Maintenance
RI: Routine Immunization
SMS: Short Message Service
SPHCDA: State Primary Health Care Development Agency
WDC: Ward Development Committee

## Footnotes

1 Attended schooling higher than the secondary level and can read a whole or part of a sentence.

2 All basic vaccinations are defined as receiving BCG, three doses of DPT-HepB-Hib, three doses of oral polio vaccine (excluding polio vaccine given at birth), and one dose of measles.

3 Socio-demographic information collected included respondent type, interview date, years in the position, years in immunization, years in health care, respondent sex, age, and education level.
