# Supplementary material for: Implementing SMS reminders for routine immunization in Northern Nigeria: a qualitative evaluation using the RE-AIM framework
Source: BMC Public Health. 2022 Dec 17;22:2370. doi: 10.1186/s12889-022-14822-1 (PMC9758467; doi:10.1186/s12889-022-14822-1)
Supplement: Supplementary file 2 — Additional file 2. Interview Guides. [file 12889_2022_14822_MOESM2_ESM.docx]

**Additional File 2.** Interview Guides

**Evaluating SMS messaging for immunization demand generation in Nigeria: An Immunization Reminder and Information SMS System (IRISS) Project**

**JHU IRB No:** IRB00008732

**NHREC:** NHREC/01/01/2007-29/08/2019B

**Focus Group Discussion Guide for male and female youth, parents of children under-five, opinion leaders, and WDC members in the community.**

|  | **Introduction** | According to NDHS 2018, RI coverage and demand for vaccines in Kebbi State is very poor at 11%. As much as 21% of children had never received any vaccine from the RI program, and only 6% were fully vaccinated by two years of age. Poor demand for vaccination has been attributed to lack of information and knowledge about the vaccination program. The IRISS project aims to overcome the information gap with SMS messaging and reminders to caregivers to increase vaccine demand, uptake and improve public confidence in the immunization system. |
| --- | --- | --- |
|  | **Purpose of this interview**: | This interview guide aims to:   1. Assess change in community’s knowledge or understanding about vaccination in the last one year. 2. Understand barriers to immunization uptake and social norms about vaccination in the last one year. 3. Understand mobile phone use patterns in target communities in the last one year. 4. Assess community’s awareness, receipt, perception and acceptability of IRISS post-intervention. |
|  | **Guidance to the interviewer**: | *Before the interview:*   1. Before beginning the interview, read the consent to the potential participant. 2. Encourage the potential participant to ask any clarifying questions and query him/her for understanding. 3. Ask for an oral permission to interview the participant.   *During the interview:*   1. Note: Each topic corresponds with a set of questions. 2. These questions are open-ended in nature.   *End of the interview:*   1. Thank the participants for their time. 2. Answer any remaining questions. 3. Provide the contact information if they have any questions or concerns:  - Dr. Chizoba Wonodi, Principal Investigator   Email: [cwonodi1@jhsph.edu](mailto:cwonodi1@jhsph.edu) or chizobabw@gmail.com   - Chisom Obi-Jeff, Project Coordinator   Email: [chisom.obi@dclnigeria.com](mailto:chisom.obi@dclnigeria.com) |

**Introduction of Participants:**

So, let’s get started by getting to know one another a little better… I’d like to go around and have everyone introduce themselves to the group.

*[Start by introducing yourself in a friendly way to help everyone relax and get to know each other better.]*

**Knowledge about vaccination**

| **S/N** | **Main question** | **Probes** |
| --- | --- | --- |
| 1.  1. | What has changed about your understanding of vaccination in the last one year? Provide examples | 1. *What about the:*     1. *Schedule i.e. vaccine a child should receive at birth, 6weeks, 10weeks, 14weeks and 9months of age*    2. *Diseases vaccines protect against*    3. *Side effects of vaccination*    4. *Benefits of vaccination Provide examples for all* 2. *What contributed to this change, if any? Provide examples* |
| 2. | How do community members get information about childhood vaccination in the last one year? | 1. *Which of the source(s) do you value the most and why? Provide examples* 2. *What has been the notable challenge on getting information about your child’s vaccination in the last one year, if any?* 3. *What do town announcers in your community do as it relates to health if any?* 4. *What type of information on immunization do they announce?*    1. *How often do they make these announcements?* ***Please specify the number of times***    2. *When was the last time he made an announcement?* ***Please specify days of the week too***    3. *Who employs or pays town announcers?* |
| 3. | In the last one year, how do community members know when to vaccinate their children in your community? | 1. *What about where to vaccinate their children?* 2. *What has been the notable challenge on getting information about where to vaccinate their children?* 3. *If they know vaccination are free (provide example if they have been charged for vaccination)* |

**Barriers to immunization uptake and social norms about vaccination**

| **S/N** | **Main question** | **Probes** |
| --- | --- | --- |
| 4. | What do/did people say about vaccination in your community in the last one year, if any? | *Provide examples* |
| 5. | What has changed about your opinion of vaccinating children in the last one year if any? Provide examples | 1. *If they think vaccination is beneficial for their children and others in the community? Why or why not?* 2. *If they think it’s necessary, why or why not?* 3. *If they think it’s the appropriate thing to do, why and why not?* 4. *If they think there will be consequences if the child is not vaccinated. Why or why not?* 5. *If they think vaccines are safe. Why or why not?* 6. *If they think vaccination is expensive (transportation cost, opportunity cost, productivity cost etc.)* 7. *If they think it is the child’s right? Why or why not?* |
| 6. | What concerns/ worries does the community have about vaccination in the last one year if any? Provide examples | *Concerns such as ignorance on the benefit of vaccination, unaware of the need for vaccination, lack of knowledge about vaccination, husband disapproval, no faith in immunization, religious concern, mothers forgot, adverse effects following immunization (AEFI), the health worker, availability of health services etc. Provide examples* |
| 7. | How well do people trust the health system (including health workers) and vaccines in your community in the last one year? Provide examples | 1. *Any reasons for that (whether trusted or not), provide examples* 2. *What about information about vaccination? Any reason for that and provide examples* |
| 8. | How willing are you to visit the health facility (HF) for your child’s vaccination in the last one year? Provide examples | 1. *Any reason for that?* 2. *What about a situation where the mother cannot visit the HF, who takes the child for vaccination?* |
| 9. | Why do mothers not vaccinate their children on time and completely in your community. What could be the reason? | 1. *What about the most common reasons children don’t get vaccinated in your community in the last one year? Provide examples* 2. *What are some of the challenges you have encountered when attempting to vaccinate your children (e.g. closed clinic, vaccine stock out etc.) in the last one year? Provide examples* |
| 10. | What is the best way to motivate community members to take their children for vaccination on time and completely? | *What about immediately after birth?* |
| 11. | Are there any specific religious, cultural or traditional practices in the last one year that prevent mothers from accessing and vaccinating their children on time and completely? | 1. *What is the practice?* 2. *How strongly are these practices adhered to when it comes to immunization in your community?* 3. *How does it influence immunization uptake? Provide examples* |
| 12. | In the last one year, how do you promote immunization in your community? Provide examples | 1. *What specific role did you play?* 2. *What about support in accessing vaccination?* 3. *Any pressure to vaccinate children in the community, and from who? Provide examples* |
| 13 | In the last one year, what influenced your decision to promote immunization in your community? Provide examples | 1. *What role did you play in supporting decision-making about childhood vaccination if any? Provide example* |
| 14 | How involved are community members in health-related activities in your community in the last one year? | *Most especially in:*   - 1. *Vaccination-related events or activities in the community such as health talks, compound meetings, etc.*   2. *Intervention design such as involvement in focus group discussions, surveys, interviews to pilot an intervention design*   3. *Programme delivery as a volunteer, peer support provider or other*   4. *Research such as participation in a study, trial or evaluation*   5. *Health care policy making such as decision making as it relates to health*   6. *Any stages of a community-directed coalition or programme*   7. *Advisory group of a community coalition or programme*   8. *Community health programme management*   *Provide example for any activity involved* |
| 15 | In the last one year, what influenced your decision to vaccinate your child and why? | 1. *Provide examples* 2. *For those that have not vaccinated, what can influence their decision to vaccinate?* |
| 16. | In the last one year, what has been the changes you have observed about vaccination in your community, if any? Provide examples | 1. *Among these changes mentioned, which of them has been the most significant? Provide examples* |

**IRISS implementation**

IRISS also known as Tunatar da ni is an intervention that use SMS to inform and educate caregivers/parents about the importance of immunization; and remind them of their child’s immunization schedules, including health facility vaccination schedules and service availability in their locality. IRISS will deliver these immunization messages in 3 ways:

1. **General broadcasts** to the public with informative messages about immunization in general and advertisements for parents to opt-in to IRISS e-registry.
2. **Targeted broadcasts** to the community gatekeepers (District Heads, Village Heads, Imams) with messages on the importance of immunization and immunization clinic days of the health centers within the ward that offer RI.
3. **Individualized reminders** of child’s vaccination schedule to caregivers who register into IRISS e-registry

During this section, we would want to also assess the awareness, reach, acceptability and perceptions about Tunatar dani. We will also assess the attitudes and actions taken after receiving the messages.

| **S/N** | **Main question** | **Probes** |
| --- | --- | --- |
| 17. | This intervention requires a phone. In the last one year, do you have regular access to a mobile phone that can be used to receive phone calls and text messages? | 1. *If you have access to a mobile phone:*    1. *Do you use it regularly?*    2. *Do you know how to check text messages on the phone?*    3. *Can you read the text messages that have been sent to you?*    4. *If you get a message and cannot read it, what will you do?*    5. *What will make you pay attention or open a text message sent to you?* 2. *If you don’t have access to a mobile phone, is there anybody in your compound that own a phone?*    1. *If you don’t have access to a mobile phone, how best can Tunatar dani send message to you* |
| 18. | Have you heard of Tunatar dani? | 1. *If yes, how did you hear about Tunatar da ni?* 2. *What is Tunatar da ni about?* 3. *What do you think about Tunatar da ni? (Provide examples)* |
| 19. | Have you received any messages on immunization? | *If so, what does the message say?* |
| 20. | Have you ever received any text messages on the days health facilities in your community will hold sessions? | 1. ***If yes:*** 2. *From who?* 3. *How often do you receive these messages?* ***Please specify the number of times*** 4. *When was the last time you got a text message on the importance of immunizations?* ***Please specify days of the week too*** 5. *What was the last message asking you to do?* 6. *When you received the message, what did you do?* 7. ***If no:***    1. *How do you know the immunization clinic days in the health center near you? Provide examples*    2. *How do you find out about the immunization clinic days at the nearest health center? Provide examples* |
| 21. | Have you ever received any text messages on the importance of immunization? | 1. *If yes,*    1. *From who?*    2. *How often do you receive these messages?* ***Please specify the number of times***    3. *When was the last time you got a text message on the importance of immunizations?* ***Please specify days of the week too***    4. *What was the last message asking you to do?*    5. *When you received the message, what did you do?* |
| 22. | Have you ever received a text message on the importance of immunization that asked you to text 'Yes' for more information? | 1. *If yes:* 2. *When you received the message what did you do?* 3. *Why did you respond to the message?* 4. *If no, why not?* |
| 23 | What new information about immunizations did you learn from Tunatar dani messages that you did not know before you got the message? | 1. *Provide examples if any* |
| 24. | What are your views about Tunatar da ni on vaccination programs? Provide examples | 1. *Is it needed and why is it needed?* 2. *Any concern and how can it be or was it addressed?* 3. *If people don’t have access to a mobile phone, how best can we send message to them?* 4. *What will make people pay attention or open a text message sent to them?* 5. *What aspects/components do you think should be or should have been included in the Tunatar da ni intervention?* 6. *What do you think should be or should have been included in the SMS reminders that would be vital for (i) caregivers (ii)health workers* |
| 25. | Who in your community have you heard that receives messages from Tunatar dani, if any? | 1. *Did you discuss the message with them to know how they feel about the messages?*    1. *What do they think about receiving text messages about immunizations, including those from Tunatar da ni? (Provide examples)*    2. *Do they think it is needed and why is it needed?* 2. *How important is it to you? Provide examples* 3. *Are vaccination SMS reminders culturally acceptable in your community, if not why?* 4. *Why would you recommend other parents to register their children for Tunatar da ni text messages? If any?* |
| 26. | One year ago, we trained community gatekeepers (i.e. District Heads, Village Heads and Mai-ungwas), health workers and LGA program managers (including community engagement focal persons at LGA and Ward levels) to help mothers that cannot read and write register for Tunatar dani to receive personalized information about their child’s vaccination due dates. What is your view about this? | 1. *Is there another approach we can use or would have used to help mothers register for Tunatar dani?* 2. *What will motivate or would have motivated mothers in the community to register for Tunatar dani?* 3. *How can we improve Tunatar da ni to increase registrations in the community?* |
| 27. | **For Intervention LGAs:** Was there any reason why caregivers/parents in your LGA did not like to receive Tunatar da ni messages? Provide examples  **For Control LGAs:** Is there any reason why community members will not like to receive these messages? | 1. *How was it or can it be addressed?* 2. *Who should have been or should be involved to improve acceptance?* 3. *What should have been or is culturally appropriate to improve acceptance?* |

**Evaluating SMS messaging for immunization demand generation in Nigeria: An Immunization Reminder and Information SMS System (IRISS) Project**

**JHU IRB No:** IRB00008732

**NHREC:** NHREC/01/01/2007-29/08/2019B

**In-depth Interview Guide for traditional and religious leaders**

The interview will be held with the traditional (Sarikis) and religious (Imans) in the community.

|  | **Introduction** | According to NDHS 2018, RI coverage and demand for vaccines in Kebbi State is very poor at 11%. As much as 21% of children had never received any vaccine from the RI program, and only 6% were fully vaccinated by two years of age. Poor demand for vaccination has been attributed to lack of information and knowledge about the vaccination program. The IRISS project aims to overcome the information gap with SMS messaging and reminders to caregivers to increase vaccine demand and uptake and improve public confidence in the immunization system. |
| --- | --- | --- |
|  | **Purpose of this interview**: | This interview guide aims to:   1. Assess change in community’s knowledge or understanding about vaccination in the last one year. 2. Understand barriers to immunization uptake and social norms about vaccination in the last one year. 3. Understand mobile phone use patterns in target communities in the last one year. 4. Assess community’s awareness, receipt, perception and acceptability of IRISS post-intervention. |
|  | **Guidance to the interviewer**: | *Before the interview:*   1. Before beginning the interview, read the consent to the potential participant. 2. Encourage the potential participant to ask any clarifying questions and query him/her for understanding. 3. Ask for an oral permission to interview the participant.   *During the interview:*   1. Note: Each topic corresponds with a set of questions. 2. These questions are open-ended in nature.   *End of the interview:*   1. Thank the participants for their time. 2. Answer any remaining questions. 3. Provide the contact information if they have any questions or concerns:  - Dr. Chizoba Wonodi, Principal Investigator   Email: [cwonodi1@jhsph.edu](mailto:cwonodi1@jhsph.edu) or chizobabw@gmail.com   - Chisom Obi-Jeff, Project Coordinator   Email: [chisom.obi@dclnigeria.com](mailto:chisom.obi@dclnigeria.com) |

**Knowledge about vaccination**

| **S/N** | **Main question** | **Probes** |
| --- | --- | --- |
| 1.  1. | What has changed about your understanding of vaccination in the last one year? Provide examples | 1. *What about the:*     1. *Schedule i.e. vaccine a child should receive at birth, 6weeks, 10weeks, 14weeks and 9months of age*    2. *Diseases vaccines protect against*    3. *Side effects of vaccination*    4. *Benefits of vaccination Provide examples for all* 2. *What contributed to this change, if any? Provide examples* |
| 2. | How do community members get information about childhood vaccination in the last one year? | 1. *Which of the source(s) do you value the most and why? Provide examples* 2. *What has been the notable challenge on getting information about your child’s vaccination in the last one year, if any?* 3. *What do town announcers in your community do as it relates to health if any?* 4. *What type of information on immunization do they announce?*    1. *How often do they make these announcements?* ***Please specify the number of times***    2. *When was the last time he made an announcement?* ***Please specify days of the week too***    3. *Who employs or pays town announcers?* |
| 3. | In the last one year, how do community members know when to vaccinate their children in your community? | 1. *What about where to vaccinate their children?* 2. *What has been the notable challenge on getting information about where to vaccinate their children?* 3. *If they know vaccination are free (provide example if they have been charged for vaccination)* |

**Barriers to immunization uptake and social norms about vaccination**

| **S/N** | **Main question** | **Probes** |
| --- | --- | --- |
| 4. | What do/did people say about vaccination in your community in the last one year if any? | *Provide examples* |
| 5. | What has changed about your perception of vaccinating children in the last one year if any? Provide examples | 1. *If they think vaccination is beneficial for their children and others in the community? Why or why not?* 2. *If they think it’s necessary, why or why not?* 3. *If they think it’s the appropriate thing to do, why and why not?* 4. *If they think there will be consequences if the child is not vaccinated. Why or why not?* 5. *If they think vaccines are safe. Why or why not?* 6. *If they think vaccination is expensive (transportation cost, opportunity cost, productivity cost etc.)* 7. *If they think it is the child’s right? Why or why not?* |
| 6. | What concerns/ worries does the community have about vaccination in the last one year if any? Provide examples | *Concerns such as ignorance on the benefit of vaccination, lack of knowledge about vaccination, husband disapproval, no faith in immunization, religious concern, mothers forgot, adverse effects following immunization (AEFI), the health worker, availability of health services etc. Provide examples* |
| 7. | How well do people trust the health system (including health workers) and vaccines in your community in the last one year? Provide examples | 1. *Any reasons for that (whether trusted or not), provide examples* 2. *What about information about vaccination? Any reason for that and provide examples* |
| 8. | How willing are you to visit the health facility (HF) for your child’s vaccination in the last one year? Provide examples | 1. *Any reason for that?* 2. *What about a situation where the mother cannot visit the HF, who takes the child for vaccination?* |
| 9. | Why do mothers not vaccinate their children on time and completely in your community. What could be the reason? | 1. *What about the most common reasons children don’t get vaccinated in your community in the last one year? Provide examples* 2. *What are some of the challenges you have encountered when attempting to vaccinate your children (e.g. closed clinic, vaccine stock out etc.) in the last one year Provide examples* |
| 10. | What is the best way to motivate community members to take their children for vaccination on time and completely? | *What about immediately after birth?* |
| 11. | Are there any specific religious, cultural or traditional practices in the last one year that prevent mothers from accessing and vaccinating their children on time and completely? | 1. *What is the practice?* 2. *How strongly are these practices adhered to when it comes to immunization in your community?* 3. *How does it influence immunization uptake? Provide examples* |
| 12. | In the last one year, how do you promote immunization in your community? Provide examples | 1. *What specific role did you play?* 2. *What about support in accessing vaccination?* 3. *Any pressure to vaccinate children in the community, and from who? Provide examples* |
| 13 | In the last one year, what influenced your decision to promote immunization in your community? Provide examples | 1. *What role did you play in supporting decision-making about childhood vaccination if any? Provide example* |
| 14 | How involved are community members in health-related activities in your community in the last one year? | *Most especially in:*   1. *Vaccination-related events or activities in the community such as health talks, compound meetings, etc.* 2. *Intervention design such as involvement in focus group discussions, surveys, interviews to pilot an intervention design* 3. *Programme delivery as a volunteer, peer support provider or other* 4. *Research such as participation in a study, trial or evaluation* 5. *Health care policy making such as decision making as it relates to health* 6. *Any stages of a community-directed coalition or programme* 7. *Advisory group of a community coalition or programme* 8. *Community health programme management*   *Provide example for any activity involved* |
| 15 | In your opinion, what influences decision to vaccinate children in your community in the last one year and why? | 1. *Provide examples* 2. *For those that have not vaccinated, what can influence their decision to vaccinate?* |
| 16. | In the last one year, what has been the changes you have observed about vaccination in your community, if any? Provide examples | 1. *Among these changes mentioned, which of them has been the most significant? Provide examples* |

**IRISS implementation**

IRISS also known as Tunatar da ni is an intervention that use SMS to inform and educate caregivers/parents about the importance of immunization; and remind them of their child’s immunization schedules, including health facility vaccination schedules and service availability in their locality. IRISS delivered these immunization messages in 3 ways:

1. **General broadcasts** to the public with informative messages about immunization in general and advertisements for parents to opt-in to IRISS e-registry.
2. **Targeted broadcasts** to the community gatekeepers (District Heads, Village Heads, Imams) with messages on the importance of immunization and immunization clinic days of the health centers within the ward that offer RI.
3. **Individualized reminders** of child’s vaccination schedule to caregivers who register into IRISS e-registry

During this section, we would want to also assess the awareness, reach, acceptability and perceptions about Tunatar dani. We will also assess the attitudes and actions taken after receiving the messages.

| **S/N** | **Main question** | **Probes** |
| --- | --- | --- |
| 17. | This intervention requires a phone. In the last one year, do you have regular access to a mobile phone that can be used to receive phone calls and text messages? | 1. *If you have access to a mobile phone:*    1. *Do you use it regularly?*    2. *Do you know how to check text messages on the phone?*    3. *Can you read the text messages that have been sent to you?*    4. *If you get a message and cannot read it, what will you do?*    5. *What will make you pay attention or open a text message sent to you?* 2. *If you don’t have access to a mobile phone, is there anybody in your compound that own a phone?*    1. *If you don’t have access to a mobile phone, how best can Tunatar da ni send message to you* |
| 18. | Have you heard of Tunatar dani? | 1. *If yes, how did you hear about Tunatar da ni?* 2. *What is Tunatar da ni about?* 3. *What do you think about Tunatar da ni? (Provide examples)* |
| 19. | Have you received any messages on immunization? | 1. *If so, what does the message say?* |
| 20. | Have you ever received any text messages on the days health facilities in your community will hold sessions? | 1. ***If yes:*** 2. *From who?* 3. *How often do you receive these messages?* ***Please specify the number of times*** 4. *When was the last time you got a text message on the importance of immunizations?* ***Please specify days of the week too*** 5. *What was the last message asking you to do?* 6. *When you received the message, what did you do?* 7. ***If no:*** 8. *How do you know the immunization clinic days in the health center near you? Provide examples* 9. *How do you find out about the immunization clinic days at the nearest health center? Provide examples* |
| 21. | Have you ever received any text messages on the importance of immunization? | 1. *If yes,* 2. *From who?* 3. *How often do you receive these messages?* ***Please specify the number of times*** 4. *When was the last time you got a text message on the importance of immunizations?* ***Please specify days of the week too*** 5. *What was the last message asking you to do?* 6. *When you received the message, what did you do?* |
| 22. | Have you ever received a text message on the importance of immunization that asked you to text 'Yes' for more information? | 1. *If yes:* 2. *When you received the message what did you do?* 3. *Why did you respond to the message?* 4. *If no, why not?* |
| 23 | What new information about immunizations did you learn from Tunatar dani messages that you did not know before you got the message? | 1. *Provide examples if any* |
| 24. | What are your views about Tunatar da ni on vaccination programs? Provide examples | 1. *Is it needed and why is it needed?* 2. *Any concern and how can it be or was it addressed?* 3. *How important is it to you? Provide examples* 4. *Are vaccination SMS reminders culturally acceptable in your community, if not why?* 5. *If people don’t have access to a mobile phone, how best can we send message to them?* 6. *What will make people pay attention or open a text message sent to them?* 7. *What aspects/components do you think should be or should have been included in the Tunatar da ni intervention?* 8. *What do you think should be or should have been included in the SMS reminders that would be vital for (i) caregivers (ii)health workers* |
| 25. | Who in your community have you heard that receives messages from Tunatar dani, if any? | 1. *Did you discuss the message with them to know how they feel about the messages?*    1. *What do they think about receiving text messages about immunizations, including those from Tunatar da ni? (Provide examples)*    2. *Do they think it is needed and why is it needed?* 2. *Why would you recommend other parents to register their children for Tunatar da ni text messages? If any?* |
| 26. | One year ago, we trained community gatekeepers (i.e. District Heads, Village Heads and Mai-ungwas), health workers and LGA program managers (including community engagement focal persons at LGA and Ward levels) to help mothers that cannot read and write register for Tunatar da ni to receive personalized information about their child’s vaccination due dates. What is your view about this? | 1. *Is there another approach we can use or would have used to help mothers register for Tunatar da ni?* 2. *What will motivate or would have motivated mothers in the community to register for Tunatar da ni?* 3. *How can we improve Tunatar da ni to increase registrations in the community?* |
| 27. | **For Intervention LGAs:** Was there any reason why caregivers/parents in your LGA did not like to receive Tunatar da ni messages? Provide examples  **For Control LGAs:** Is there any reason why community members will not like to receive these messages? | 1. *How was it or can it be addressed?* 2. *Who should have been or should be involved to improve acceptance?* 3. *What should have been or is culturally appropriate to improve acceptance?* |

**Evaluating SMS messaging for immunization demand generation in Nigeria: An Immunization Reminder and Information SMS System (IRISS) Project**

**JHU IRB No:** IRB00008732

**NHREC:** NHREC/01/01/2007-29/08/2019B

**In-depth interview guide for pregnant women, parents of newborns, parents of children under-five years of age, and young men and women in the community.**

|  | **Introduction** | According to NDHS 2018, RI coverage and demand for vaccines in Kebbi State is very poor at 11%. As much as 21% of children had never received any vaccine from the RI program, and only 6% were fully vaccinated by two years of age. Poor demand for vaccination has been attributed to lack of information and knowledge about the vaccination program. The IRISS project aims to overcome the information gap with SMS messaging and reminders to caregivers to increase vaccine demand and uptake and improve public confidence in the immunization system. |
| --- | --- | --- |
|  | **Purpose of this interview**: | This interview guide aims to:   1. Assess change in parent’s knowledge or understanding about vaccination in the last one year. 2. Understand barriers to immunization uptake and social norms about vaccination in the last one year. 3. Understand mobile phone use patterns in target communities in the last one year. 4. Assess community’s awareness, receipt, perception and acceptability of IRISS post-intervention. |
|  | **Guidance to the interviewer**: | *Before the interview:*   1. Before beginning the interview, read the consent to the potential participant. 2. Encourage the potential participant to ask any clarifying questions and query him/her for understanding. 3. Ask for an oral permission to interview the participant.   *During the interview:*   1. Note: Each topic corresponds with a set of questions. 2. These questions are open-ended in nature.   *End of the interview:*   1. Thank the participants for their time. 2. Answer any remaining questions. 3. Provide the contact information if they have any questions or concerns:  - Dr. Chizoba Wonodi, Principal Investigator   Email: [cwonodi1@jhsph.edu](mailto:cwonodi1@jhsph.edu) or chizobabw@gmail.com   - Chisom Obi-Jeff, Project Coordinator   Email: [chisom.obi@dclnigeria.com](mailto:chisom.obi@dclnigeria.com) |

**Knowledge about vaccination**

| **S/N** | **Main question** | **Probes** |
| --- | --- | --- |
| 1.  1. | What has changed about your understanding of vaccination in the last one year? Provide examples | 1. *What about the:*     1. *Schedule i.e. vaccine a child should receive at birth, 6weeks, 10weeks, 14weeks and 9months of age*    2. *Diseases vaccines protect against*    3. *Side effects of vaccination*    4. *Benefits of vaccination Provide examples for all* 2. *What contributed to this change, if any? Provide examples* |
| 2. | How do you get information about childhood vaccination in the last one year? | 1. *Which of the source(s) do you value the most and why? Provide examples* 2. *What has been the notable challenge on getting information about your child’s vaccination in the last one year if any?* 3. *What do town announcers in your community do as it relates to health if any?* 4. *What type of information on immunization do they announce?* 5. *How often do they make these announcements?* ***Please specify the number of times*** 6. *When was the last time he made an announcement?* ***Please specify days of the week too*** 7. *Who employs or pays town announcers?* |
| 3. | In the last one year, how do you know when to vaccinate their children? | 1. *What about where to vaccinate their children?* 2. *What has been the notable challenge on getting information about where to vaccinate their children?* 3. *If they know vaccination are free (provide example if they have been charged for vaccination)* |

**Barriers to immunization uptake and social norms about vaccination**

| **S/N** | **Main question** | **Probes** |
| --- | --- | --- |
| 4. | What do/did people say about vaccination in your community in the last one year, if any? | *Provide examples* |
| 5. | What has changed about your perception of vaccinating children in the last one year if any? Provide examples | 1. *If they think vaccination is beneficial for their children and others in the community? Why or why not?* 2. *If they think it’s necessary, why or why not?* 3. *If they think it’s the appropriate thing to do, why and why not?* 4. *If they think there will be consequences if the child is not vaccinated. Why or why not?* 5. *If they think vaccines are safe. Why or why not?* 6. *If they think vaccination is expensive (transportation cost, opportunity cost, productivity cost etc.)* 7. *If they think it is the child’s right? Why or why not?* |
| 6. | What concerns/ worries do you have about vaccination in the last one year if any? Provide examples | *Concerns such as ignorance on the benefit of vaccination, unaware of the need for vaccination, lack of knowledge about vaccination, husband disapproval, no faith in immunization, religious concern, mothers forgot, adverse effects following immunization (AEFI), the health worker, availability of health services etc. Provide examples* |
| 7. | How well do you trust the health system (including health workers) and vaccines in your community in the last one year? Provide examples | 1. *Any reasons for that (whether trusted or not), provide examples* 2. *What about information about vaccination? Any reason for that and provide examples* |
| 8. | How willing are you to visit the health facility (HF) for your child’s vaccination in the last one year? Provide examples | 1. *Any reason for that?* 2. *What about a situation where the mother cannot visit the HF, who takes the child for vaccination?* |
| 9. | Why do mothers not vaccinate their children on time and completely in your community. What could be the reason? | 1. *What about the most common reasons children don’t get vaccinated in your community in the last one year? Provide examples* 2. *What are some of the challenges you have encountered when attempting to vaccinate your children (e.g. closed clinic, vaccine stock out etc?) in the last one year? Provide examples* |
| 10. | What is the best way to motivate you to take your children for vaccination on time and completely? | *What about immediately after birth?* |
| 11. | Are there any specific religious, cultural or traditional practices in the last one year that prevent you from accessing and vaccinating your children on time and completely? | 1. *What is the practice?* 2. *How strongly are these practices adhered to when it comes to immunization in your community?* 3. *How does it influence immunization uptake? Provide examples* |
| 12. | In the last one year, how do community leaders (religious or political or traditional leaders) and health workers support vaccination for infants and children in your community? Provide examples | 1. *What specific role did they play?* 2. *What about support in accessing vaccination?* 3. *Any pressure to vaccinate children in the community, and from who? Provide examples* |
| 13 | In the last one year, what influenced your decision to promote immunization in your community if any? Provide examples | 1. *What role did you play in supporting decision-making about childhood vaccination if any? Provide examples* |
| 14 | How involved are you in health-related activities in your community in the last one year? | *Most especially in:*   1. *Vaccination-related events or activities in the community such as health talks, compound meetings, etc.* 2. *Intervention design such as involvement in focus group discussions, surveys, interviews to pilot an intervention design* 3. *Programme delivery as a volunteer, peer support provider or other* 4. *Research such as participation in a study, trial or evaluation* 5. *Health care policy making such as decision making as it relates to health* 6. *Any stages of a community-directed coalition or programme* 7. *Advisory group of a community coalition or programme* 8. *Community health programme management*   *Provide example for any activity involved* |
| 15 | In the last one year, what influenced your decision to vaccinate your child and why? | 1. *Provide examples* 2. *For those that have not vaccinated, what can influence their decision to vaccinate?* |
| 16. | In the last one year, what has been the changes you have observed about vaccination in your community, if any? Provide examples | 1. *Among these changes mentioned, which of them has been the most significant? Provide examples* |

**IRISS implementation**

IRISS also known as Tunatar da ni is an intervention that use SMS to inform and educate caregivers/parents about the importance of immunization; and remind them of their child’s immunization schedules, including health facility vaccination schedules and service availability in their locality. IRISS delivers these immunization messages in three ways:

1. **General broadcasts** to the public with informative messages about immunization in general and advertisements for parents to opt-in to IRISS e-registry.
2. **Targeted broadcasts** to the community gatekeepers (District Heads, Village Heads, Imams) with messages on the importance of immunization and immunization clinic days of the health centers within the ward that offer RI.
3. **Individualized reminders** of child’s vaccination schedule to caregivers who register into IRISS e-registry

During this section, we would want to also assess the awareness, reach, acceptability and perceptions about Tunatar dani. We will also assess the attitudes and actions taken after receiving the messages.

| **S/N** | **Main question** | **Probes** |
| --- | --- | --- |
| 17. | This intervention requires a phone. In the last one year, do you have regular access to a mobile phone that can be used to receive phone calls and text messages? | 1. *If you have access to a mobile phone:*    1. *Do you use it regularly?*    2. *Do you know how to check text messages on the phone?*    3. *Can you read the text messages that have been sent to you?*    4. *If you get a message and cannot read it, what will you do?*    5. *What will make you pay attention or open a text message sent to you?* 2. *If you don’t have access to a mobile phone, is there anybody in your compound that own a phone?*    1. *If you don’t have access to a mobile phone, how best can we send Tunatar da ni message to you* |
| 18. | Have you heard of Tunatar dani? | 1. *If yes, how did you hear about Tunatar da ni?* 2. *What is Tunatar da ni about?* 3. *What do you think about Tunatar da ni? (Provide examples)* |
| 19. | Have you received any messages on immunization? | *If so, what does the message say?* |
| 20. | Have you ever received any text messages on the days health facilities in your community will hold sessions? | 1. ***If yes:*** 2. *From who?* 3. *How often do you receive these messages?* ***Please specify the number of times*** 4. *When was the last time you got a text message on the importance of immunizations?* ***Please specify days of the week too*** 5. *What was the last message asking you to do?* 6. *When you received the message, what did you do?* 7. ***If no:*** 8. *How do you know the immunization clinic days in the health center near you? Provide examples* 9. *How do you find out about the immunization clinic days at the nearest health center? Provide examples* |
| 21. | Have you ever received any text messages on the importance of immunization? | *If yes,*   1. *From who?* 2. *How often do you receive these messages?* ***Please specify the number of times*** 3. *When was the last time you got a text message on the importance of immunizations?* ***Please specify days of the week too*** 4. *What was the last message asking you to do?* 5. *When you received the message, what did you do?* |
| 22. | Have you ever received a text message on the importance of immunization that asked you to text 'Yes' for more information? | 1. *If yes:* 2. *When you received the message what did you do?* 3. *Why did you respond to the message?* 4. *If no, why not?* |
| 23 | What new information about immunizations did you learn from Tunatar dani messages that you did not know before you got the message? | 1. *Provide examples if any* |
| 24. | What are your views about Tunatar da ni on vaccination programs? Provide examples | 1. *Is it needed and why is it needed?* 2. *How important is it to you? Provide examples* 3. *Are vaccination SMS reminders culturally acceptable in your community, if not why?* 4. *Why would you recommend other parents to register their children for Tunatar da ni text messages? If any?* 5. *If people don’t have access to a mobile phone, how best can we send message to them?* 6. *What will make people pay attention or open a text message sent to them?* 7. *What aspects/components do you think should be or should have been included in the Tunatar da ni intervention?* 8. *What do you think should be or should have been included in the SMS reminders that would be vital for (i) caregivers (ii)health workers* |
| 25. | Who in your community have you heard that receives messages from Tunatar dani, if any? | 1. *Did you discuss the message with them to know how they feel about the messages?*    1. *What do they think about receiving text messages about immunizations, including those from Tunatar da ni? (Provide examples)*    2. *Do they think it is needed and why is it needed?* 2. *Why would you recommend other parents to register their children for Tunatar da ni text messages? If any?* |
| 26. | **Intervention LGAs:** What made you register for Tunatar da ni? Provide reasons  **Control LGAs:** How willing are you to register into Tunatar da ni to receive personalized information about your child’s vaccination due dates? Any reasons? | ***Intervention LGAs:***   1. *What challenges did you encounter with Tunatar da ni registrations and messages, if any? Provide examples*   ***Control LGAs:*** *Registering into IRISS to receive reminders about your child’s vaccination due dates will require that you send information about your child such as the child’s name, date of birth, name of HF and date of birth*   1. *How willing are you to pay for the SMS messages sent as part of registration for Tunatar da ni in order to receive reminders about your child’s vaccination due dates? Any reasons?* |
| 27. | One year ago, we trained community gatekeepers (i.e. District Heads, Village Heads and Mai-ungwas), health workers and LGA program managers (including community engagement focal persons at LGA and Ward levels) to help mothers that cannot read and write register into IRISS e-registry to receive personalized information about their child’s vaccination due dates. What is your view about this? | 1. *Is there another approach we can use or would have used to help mothers register for Tunatar dani?* 2. *What will motivate or would have motivated mothers in the community to register for Tunatar dani?* 3. *How can we improve Tunatar da ni to increase registrations in the community?* |
| 28. | **For Intervention LGAs:** Was there any reason why caregivers/parents in your LGA did not like to receive Tunatar da ni messages? Provide examples  **For Control LGAs:** Is there any reason why community members will not like to receive these messages? | 1. *How was it or can it be addressed?* 2. *Who should have been or should be involved to improve acceptance?* 3. *What should have been or is culturally appropriate to improve acceptance?* |

**Evaluating SMS messaging for immunization demand generation in Nigeria: An Immunization Reminder and Information SMS System (IRISS) Project**

**JHU IRB No:** IRB00008732

**NHREC:** NHREC/01/01/2007-29/08/2019B

**Key Informant Interview Guide for RI Providers**

The interview will be held with the Officer in-charge (OIC)/Ward focal person (WFP) or Officer in charge for routine immunization, or RI focal person.

|  | **Introduction** | According to NDHS 2018, RI coverage and demand for vaccines in Kebbi State is very poor at 11%. As much as 21% of children had never received any vaccine from the RI program, and only 6% were fully vaccinated by two years of age. Poor demand for vaccination has been attributed to lack of information and knowledge about the vaccination program. The IRISS project aims to overcome the information gap with SMS messaging and reminders to caregivers to increase vaccine demand and uptake and improve public confidence in the immunization system. |
| --- | --- | --- |
|  | **Purpose of this interview**: | This interview guide aims to:   1. Understand barriers to immunization uptake and social norms about vaccination in the last one year. 2. Understand mobile phone use patterns in target communities in the last one year. 3. Assess change in availability of RI services in the last one year. 4. Assess health worker’s role in assisting mothers and gatekeepers register on IRISS 5. Determine the human, material and financial resources needed to implement a similar program at state level 6. Assess the impact of COVID on service delivery during IRISS intervention |
|  | **Guidance to the interviewer**: | *Before the interview:*   1. Before beginning the interview, read the consent to the potential participant. 2. Encourage the potential participant to ask any clarifying questions and query him/her for understanding. 3. Ask for an oral permission to interview the participant.   *During the interview:*   1. Note: Each topic corresponds with a set of questions. 2. These questions are open-ended in nature.   *End of the interview:*   1. Thank the participants for their time. 2. Answer any remaining questions. 3. Provide the contact information if they have any questions or concerns:  - Dr. Chizoba Wonodi, Principal Investigator   Email: [cwonodi1@jhsph.edu](mailto:cwonodi1@jhsph.edu) or chizobabw@gmail.com   - Chisom Obi-Jeff, Project Coordinator   Email: [chisom.obi@dclnigeria.com](mailto:chisom.obi@dclnigeria.com) |

**Barriers to immunization uptake and social norms about vaccination.**

| **S/N** | **Main question** | **Probes** |
| --- | --- | --- |
| 1. | According to Q3 2019 LQAS, RI coverage in this LGA was low. What could be the reason? | 1. *What about the most common reasons mothers do not vaccinate their children on time and completely in this community?* 2. *As the health worker, are there other reasons you think that lead to low RI coverage in your LGA?* 3. *How has this changed or remained the same in the last one year. Provide examples* |
| 2. | What is the best way to motivate caregivers/mothers to take their children for vaccination on time and completely? | *What about immediately after birth?* |
| 3. | Are there any specific religious, cultural or traditional practices in the last one year that prevent mothers from accessing and vaccinating their children on time and completely? | 1. *What is the practice?* 2. *How strongly are these practices adhered to when it comes to immunization in your community?* 3. *How does it influence immunization uptake in your HF? Provide examples* 4. *What about attendance for immunization days, dropout, coverage etc.* |
| 4. | In the last one year, how do you encourage vaccination for infants and children in the community? Provide examples | 1. *What specific role did you play?* 2. *What role did you play in supporting decision-making about childhood vaccination? Provide examples* 3. *What about support in accessing vaccination?* 4. *Any pressure to vaccinate children in the community, from who and what direction? Provide examples*    1. *What about from SERICC, LERICC etc.*    2. *What about supportive supervision* |
| 5. | In the last one year, what factors influenced your decision to promote immunizations in your community? | *Provide examples* |

**Availability of RI services**

| **S/N** | **Main question** | **Probes** |
| --- | --- | --- |
| 6. | What has disrupted RI services in your HF in the last one year, if any? Provide examples | 1. *Any vaccine stock outs and other RI commodities e.g AD syringes* 2. *Do health workers conduct their fixed and outreach sessions as stated in their microplans, If not why?* |
| 7. | What are the major challenges in delivering RI services in this HF in the last one year? | 1. ***Supply side challenges:*** 2. *Funding for RI activities,* 3. *Availability of cold chain equipment and health workers to conduct the RI sessions,* 4. *Health systems governance: How is it managed at the state, LGA and HF level? If the issue is to be fixed, what will it take? Who do we need to fix the issue?* 5. ***Demand side challenges:*** 6. *Community opposition to vaccination,* 7. *Immunization services not getting to community members* 8. *Lack of knowledge or empowerment to demand RI services from community members* 9. *Poor community engagement* |
| 8. | How best can availability of RI services in this HF be improved? |  |
| 9 | In the three months (i.e. since Feb. 2020), how the conduct of fixed and outreach sessions in your HF been compared to the whole of last year? Provide examples | 1. *What is responsible for the difference?* 2. *How has it affected the conduct of fixed and outreach sessions in your HF?* |
| 10. | How has COVID affected your ability to provide vaccination services for fixed and outreach sessions? Provide examples | 1. *Has there been a disruption? If yes, why?* 2. *Have you been holding services? If no, what are the reasons* 3. *Have community members been coming to receive RI? If no, why?* 4. *How has lockdown, travel restrictions, supply shortages etc. affected availability of RI services in your HF? Provide examples* |

**IRISS implementation**

IRISS also known as Tunatar da ni is an intervention that use SMS to inform and educate caregivers/parents about the importance of immunization; and remind them of their child’s immunization schedules, including health facility vaccination schedules and service availability in their locality. IRISS delivers these immunization messages in three ways:

1. **General broadcasts** to the public with informative messages about immunization in general and advertisements for parents to opt-in to IRISS e-registry.
2. **Targeted broadcasts** to the community gatekeepers (District Heads, Village Heads, Imams) with messages on the importance of immunization and immunization clinic days of the health centers within the ward that offer RI.
3. **Individualized reminders** of child’s vaccination schedule to caregivers who register into IRISS e-registry

| **S/N** | **Main question** | **Probes** |
| --- | --- | --- |
| 11. | Have you heard of Tunatar dani? | 1. *If yes, how did you hear about Tunatar da ni?* 2. *What is Tunatar da ni about?* 3. *Have you ever received any text messages from Tunatar da ni? If yes, what is the message about?* |
| 12. | Have you received any messages on immunization? | 1. *If so, what does the message say?* |
| 13. | Have you ever received any text messages on the days health facilities in your community will hold sessions?    E.g. | 1. ***If yes:*** 2. *From who?* 3. *How often do you receive these messages?* ***Please specify the number of times*** 4. *When was the last time you got a text message on the importance of immunizations?* ***Please specify days of the week too*** 5. *What was the last message asking you to do?* 6. *When you received the message, what did you do? (Respondent show message from their phone)* 7. ***If no:*** 8. *How do you know the immunization clinic days in the health center near you? Provide examples* 9. *How do you find out about the immunization clinic days at the nearest health center? Provide examples* |
| 14. | Have you ever received any text messages on the importance of immunization?  E.g. | 1. *If yes,* 2. *From who?* 3. *How often do you receive these messages?* ***Please specify the number of times*** 4. *When was the last time you got a text message on the importance of immunizations?* ***Please specify days of the week too*** 5. *What was the last message asking you to do? (Respondent show message from their phone)* 6. *When you received the message, what did you do?* |
| 15. | Have you ever received a text message on the importance of immunization that asked you to text 'Yes' for more information?  E.g. | 1. *If yes:* 2. *When you received the message what did you do?* 3. *Why did you respond to the message?* 4. *If no, why not?* |
| 16. | Have you ever received any text message on COVI9 that asked you to text 'Yes' for more information? | 1. *If yes:* 2. *When you received the message what did you do?* 3. *Why did you respond to the message?* 4. *If no, why not?* |
| 17. | What are your views about Tunatar da ni on vaccination programs? Provide examples | 1. *Is it needed and why is it needed?* 2. *Any concern and how can it be or was it addressed?* 3. *If people don’t have access to a mobile phone, how best can we send message to them?* 4. *What will make people pay attention or open a text message sent to them?* 5. *What aspects/components do you think should be or should have been included in the Tunatar da ni intervention?* 6. *What do you think should be or should have been included in the SMS reminders that would be vital for (i) caregivers (ii)health workers (iii) program managers* |
| 18. | Who in your community have you heard that receives messages from Tunatar dani, if any? | 1. *Did you discuss the message with them to know how they feel about the messages?*    1. *What do they think about receiving text messages about immunizations, including those from Tunatar da ni? (Provide examples)*    2. *Do they think it is needed and why is it needed?* 2. *How important is it to you? Provide examples* 3. *Are vaccination SMS reminders culturally acceptable in your community, if not why?* 4. *Why would you recommend other parents to register their children for Tunatar da ni text messages? If any?* |
| 19. | **For Intervention LGAs:** How did you advocate for parents to register for Tunatar da ni to receive personalized information about their child’s vaccination due dates? Provide examples  **For Control LGAs:** How willing are you to advocate for parents to register into IRISS e-registry to receive personalized information about their child’s vaccination due dates? | 1. *Any reason for that?* 2. *Any reason for not willing to advocate?* 3. *What in your opinion will motivate or did not motivate mothers in the community to register for Tunatar da ni?* 4. *Any pressure to register children in the community, from who and what direction? Provide examples* |
| 20. | **For Intervention LGAs:** When do you register children more? Provide examples | 1. *If you met 10 caregivers, how many will have a phone or access to a phone?*     1. *How many will know the phone number for you to use to register their child?* 2. *If you have a total of 10 children registered. How many will you say registered during fixed vs outreach sessions?* |
| 21. | **For Intervention LGAs:**  You registered X number of children in the last one year to receive reminders with your phone, what were the reasons why you used your phone? | 1. *We have a list of reasons why RI providers register their own phones for reminders instead of the caregiver’s phone. Which one of these are the main reasons:*    1. *Pressure from supportive supervision to register more children*    2. *To serve as a defaulter tracker for RI provider*    3. *Mothers do not remember husband’s phone number*    4. *Caregivers cannot read or type text messages*    5. *Caregivers do not have phones*    6. *Incentives from proxy registration*    7. *Poor network around catchment settlement* 2. *When you received the reminders what did you do? Provide examples*    1. *How were you able to remind that number of children about their due dates?*    2. *What actions did the caregiver take? Provide examples* 3. *Why were parents not registering for Tunatar da ni themselves?* 4. *What has changed or did not change in terms of utilization of RI services since Tunatar da ni? Provide examples* |
| 22. | **For Intervention LGAs:** How many gatekeepers did you register to receive Tunatar da ni messages (i.e messages on the importance of immunization and dates immunization services will hold in your HF) on your phone? | 1. *Why were you registering these gatekeepers?*    1. *Provide examples on the reasons for registering these gatekeepers*    2. *Why were gatekeepers not registering for Tunatar da ni themselves?*    3. *What has changed or did not change in terms of utilization of RI services since Tunatar da ni? Provide examples* 2. *When you received the message what did you do? Provide examples* 3. *What actions did the gatekeeper take? Provide examples* |
| 23. | One year ago, we trained community gatekeepers (i.e. District Heads, Village Heads and Mai-ungwas), health workers and LGA program managers (including community engagement focal persons at LGA and Ward levels) to help mothers that cannot read and write register for Tunatar da ni to receive personalized information about their child’s vaccination due dates. What is your view about this? | 1. *What can motivate you or motivated you to provide this service to parents in this community?* 2. *What potential barriers would you or did you face in providing this service, and how can it be or was it mitigated* 3. *Is there another approach we can use or would have used to help mothers register for Tunatar da ni?* 4. *How can we improve Tunatar da ni to increase registrations in the community?* |
| 24. | **For Intervention LGAs:** Was there any reason why caregivers/parents in your LGA did not like to receive Tunatar da ni messages? Provide examples  **For Control LGAs:** Is there any reason why caregivers/parents will not like to receive these messages? | 1. *How was or can it be addressed?* 2. *Who should have been or should be involved to improve acceptance?* 3. *What should have been or is culturally appropriate to improve acceptance?* |
| 25. | What programs have happened in your community that may have an impact on immunization, if any | 1. *What contributed to the success of the intervention? Provide examples* 2. *What were the challenges?* 3. *What were the lessons learnt?* 4. *What interventions have happened for COVID? Please describe* |

**Health Worker Feedback** *(Completed among health workers in intervention LGAs: Fakai and Aleiro)*

This section collects information on experiences using the IRISS system at the health facility level.

| **S/N** | **Main question** | **Probes** |
| --- | --- | --- |
| 26. | How much time per week did you spend on activities related to the Tunatar da ni intervention? Examples of Tunatar da ni activities include time spent on explaining the Tunatar da ni to other staff or caregivers, helping caregivers register into Tunatar da ni, and informing parents who you registered using your phone about their child’s immunization due dates. | 1. *Number of hours per week?* 2. *Number of hours you spend per week on Tunatar da ni related activities* 3. *Percentage of total hours worked per week?* |
| 27. | What challenges, if any, did you and other health workers faced when implementing the Tunatar da ni? | 1. *Was the Tunatar da ni* *registration difficult? Explain with examples* 2. *Was the information provided in the SMS messages correct? Explain with examples* 3. *How was the workload related to implementing Tunatar da ni? Provide examples* 4. *Were there other challenges?* |
| 28 | What do you consider the strengths of Tunatar da ni intervention? Provide examples | 1. *What was the caregiver reaction to receiving the SMS messages? Provide examples* 2. *If you have 10 caregivers who visited the HF for child’s vaccination, how many came due to Tunatar da ni reminder sent to them?* |
| 29. | Is there any other information or feedback you wish to share about the implementation of Tunatar da ni? |  |

**Evaluating SMS messaging for immunization demand generation in Nigeria: An Immunization Reminder and Information SMS System (IRISS) Project**

**JHU IRB No:** IRB00008732

**NHREC:** NHREC/01/01/2007-29/08/2019B

**Key Informant Interview Guide for Local Government Area (LGA) immunization program manager.**

The interview will be held with the LGA Routine Immunization Officer

|  | **Introduction** | According to NDHS 2018, RI coverage and demand for vaccines in Kebbi State is very poor at 11%. As much as 21% of children had never received any vaccine from the RI program, and only 6% were fully vaccinated by two years of age. This has been attributed to lack of information and knowledge about the vaccination program. The IRISS project aims to overcome the information gap with SMS messaging and reminders to caregivers to increase vaccine demand and uptake and improve public confidence in the immunization system. |
| --- | --- | --- |
|  | **Purpose of this interview**: | This interview guide aims to:   1. Understand changes in the RI program context in the last one year. 2. Evaluate availability of RI services in the last one year. 3. Assess programmatic feasibility of delivering the IRISS intervention. 4. Understand barriers to immunization uptake and social norms about vaccination in the last one year. |
|  | **Guidance to the interviewer**: | *Before the interview:*   1. Before beginning the interview, read the consent to the potential participant. 2. Encourage the potential participant to ask any clarifying questions and query him/her for understanding. 3. Ask for an oral permission to interview the participant.   *During the interview:*   1. Note: Each topic corresponds with a set of questions. 2. These questions are open-ended in nature.   *End of the interview:*   1. Thank the participants for their time. 2. Answer any remaining questions. 3. Provide the contact information if they have any questions or concerns:  - Dr. Chizoba Wonodi, Principal Investigator   Email: [cwonodi1@jhsph.edu](mailto:cwonodi1@jhsph.edu) or chizobabw@gmail.com   - Chisom Obi-Jeff, Project Coordinator   Email: [chisom.obi@dclnigeria.com](mailto:chisom.obi@dclnigeria.com) |

**RI program context**

| **S/N** | **Main question** | **Probes** |
| --- | --- | --- |
| 1. | In your view, what has been the most significant change in the RI program in the last one year? Provide examples | Any reasons? |
| 1.  2. | In the last one year, what new immunization programs were introduced in yourLGA by national, state, partners and CSOs? | 1. *What are the activities?* 2. *How many wards are covered?* 3. *What is the duration of the program?* 4. *What successes have been recorded with the program?* 5. *What challenges have been recorded with the program?* 6. *What are the lessons learnt?* 7. *Who is funding it?* 8. *What other special programs are being conducted in your LGA? Describe* |
| 3 3. | In the last one year, what new RI partners work in the LGA? | 1. *Which program do each partner support?* 2. *Which aspects of immunization programs do each partner support?* 3. *What RI services do they provide?* 4. *Are all the wards covered?* 5. *Any report or material to provide more details?* |
| 2.  4. | In the last one year, were there any programs that involve working with community leaders (eg. traditional/religious leaders), health workers, Ward CEFPs etc in the LGA? | 1. *If yes, please list them* 2. *Are they active?* 3. *What activities do they conduct?* |
| 5. | Do you have specific program where community sensitization and reminder and education on vaccination is conducted in your LGA in the last one year? | 1. *What is the name of the program?* 2. *Who organizes such program?* 3. *What does the program entail?* |

**Availability of RI services**

| **S/N** | **Main question** | **Probes** |
| --- | --- | --- |
| 6. | According to Q3 2019 LQAS, RI coverage in your LGA is low at X. What could be the reason? | 1. *Any knowledge on the most common reasons mothers do not vaccinate their children on time and completely in your LGA?* 2. *As the LGA program manager, are there other reasons you think that lead to low RI coverage in your LGA?* |
| 7. | What has disrupted RI services in your LGA in the last one year, if any? Provide examples | 1. *Any vaccine stock outs and other RI commodities e.g AD syringes* 2. *Do health workers conduct their fixed and outreach sessions as stated in their microplans, If not why?* |
| 8. | What are the major challenges in delivering RI services in your LGA in the last one year, if any? | 1. ***Supply side challenges:*** 2. *Funding for RI activities,* 3. *Availability of cold chain equipment and health workers to conduct the RI sessions,* 4. *Health systems governance: How is it managed at the state, LGA and HF level? If the issue is to be fixed, what will it take? Who do we need to fix the issue?* 5. ***Demand side challenges:*** 6. *Community opposition to vaccination,* 7. *Immunization services not getting to community members* 8. *Lack of knowledge or empowerment to demand RI services from community members* 9. *Poor community engagement* |
| 9. | How best can RI services and coverage in your LGA be improved? Provide examples | *a. What resources are needed?* |

**IRISS implementation**

IRISS also known as Tunatar da ni is an intervention that use SMS to inform and educate caregivers/parents about the importance of immunization; and remind them of their child’s immunization schedules, including health facility vaccination schedules and service availability in their locality. IRISS delivered these immunization messages in 3 ways:

1. **General broadcasts** to the public with informative messages about immunization in general and advertisements for parents to opt-in to IRISS e-registry.
2. **Targeted broadcasts** to the community gatekeepers (District Heads, Village Heads, Imams) with messages on the importance of immunization and immunization clinic days of the health centers within the ward that offer RI.
3. **Individualized reminders** of child’s vaccination schedule to caregivers who register into IRISS e-registry

| **S/N** | **Main question** | **Probes** |
| --- | --- | --- |
| 10. | Have you heard of Tunatar dani? | 1. *If yes, how did you hear about Tunatar da ni?* 2. *What is Tunatar da ni about?* 3. *Have you ever received any text messages from Tunatar da ni? If yes, what is the message about?* 4. *Who in your LGA have you heard that receives messages from Tunatar dani if any?* |
| 11. | What are your views about Tunatar da ni on vaccination programs? Provide examples | 1. *Was/Is it needed and why was/is it needed?* 2. *Any concern and how can it be addressed?* 3. *If people don’t have access to a mobile phone, how best can we send message to them?* 4. *What will make people pay attention or open a text message sent to them?* 5. *What aspects/components do you think should be or should have been included in the Tunatar da ni intervention?* 6. *What do you think should be or should have been included in the SMS reminders that would be vital for (i) caregivers, (ii)health workers (iii) program managers* |
| 12. | In the course of the project, majority of the registrations were done by RI providers who use their phone to register caregivers in order to receive the reminder on their phones. What do you feel about this on the impact of the RI program? | 1. *What are your thoughts about using Tunatar da ni for defaulter tracking? Please describe* |
| 13. | **For Intervention LGAs:** As a program manager, what are the efforts you made to ensure RI providers register caregivers for Tunatar da ni to receive personalized information about their child’s vaccination due dates?  **For Control LGAs:** How willing are you to advocate for parents to register into IRISS e-registry to receive personalized information about their child’s vaccination due dates? | 1. *Any reason for that?* 2. *Any reason for not willing to advocate?* 3. *What will or did motivate mothers in the community to register into Tunatar da ni?* |
| 14. | One year ago, we trained community gatekeepers (i.e. District Heads, Village Heads and Mai-ungwas), health workers and LGA program managers (including community engagement focal persons at LGA and Ward levels) to help mothers that cannot read and write register for Tunatar da ni to receive personalized information about their child’s vaccination due dates. What is your view about this? | 1. *What can or did motivate them to provide this service to parents?* 2. *What potential barriers would they or did they face in providing this service, and how can it be or was it mitigated?* 3. *Is there another approach we can use or would have used to help mothers register for Tunatar da ni?* |
| 15. | **For Intervention LGAs:** Was there any reason why caregivers/parents in your LGA did not like to receive Tunatar da ni messages? Provide examples  **For Control LGAs:** Is there any reason why caregivers/parents in your LGA will not like to receive these messages? | 1. *How can it be or was it addressed?* 2. *Who should be or should have been involved to improve acceptance?* 3. *What is or should have been culturally appropriate to improve acceptance if any?* |

**Barriers to immunization uptake and social norms about vaccination**

| **S/N** | **Main question** | **Probes** |
| --- | --- | --- |
| 16. | What do/did people say about vaccination in your LGA/community in the last one year? | *Provide examples* |
| 17. | What concerns/ worries does the community have about vaccination in the last one year? | *Concerns such as as ignorance on the benefit of vaccination, unaware of the need for vaccination, lack of knowledge about vaccination, husband disapproval,no faith in immunization, religious concern, mothers forgot, adverse effects following immunization (AEFI), the health worker, availability of health services etc.*  *Provide examples* |
| 18. | How well do people trust the health system (including health workers) and vaccines in your LGA/community in the last one year? Provide examples | 1. *Any reasons for that (whether trusted or not), provide examples* 2. *What about information about vaccination? Any reason for that and provide examples* |
| 19. | Why do mothers/caregivers not vaccinate their children on time and completely in your LGA/community. What could be the reason? | 1. *What about the most common reasons children don’t get vaccinated in your community in the last one year? Provide examples* 2. *What are some of the challenges they have encountered when attempting to vaccinate their children (e.g. closed clinic, vaccine stock out etc.) in the last one year? Provide examples* |
| 20. | What is the best way to motivate community members to take their children for vaccination on time and completely? | *What about immediately after birth?* |
| 21. | Are there any specific religious, cultural or traditional practices that prevent mothers from accessing and vaccinating their children on time and completely in the last one year? | 1. *What is the practice?* 2. *How strongly are these practices adhered to when it comes to immunization in your community?* 3. *How does it influence immunization uptake? Provide examples* |
| 22. | In the last one year, how do or did community leaders (religious or political or traditional leaders), health workers, support vaccination for infants and children in your LGA? Provide examples on the type of support | 1. *What specific role did they play? Provide examples* 2. *What about support in advocating or encouraging registration for Tunatar da ni* *in the last one year? Provide examples* 3. *What about support in accessing vaccination* *in the last one year? Provide examples* 4. *In the last one year, has there been any pressure to vaccinate children in the community, and from who? Provide examples* |
| 23 | In the last one year, how did you promote immunization in your LGA? Provide examples | 1. *What specific role did you play?* 2. *What about support in accessing vaccination?* 3. *What about supporting community engagement in the last one year? Provide example* 4. *Any pressure to vaccinate children in the community, and from who? Provide examples* |
| 24. | In the last one year, what influenced your decision to promote immunization in your LGA? | 1. *Provide examples* |
| 25. | How involved are community members in health-related activities in your LGA/community in the last one year? | *Most especially in:*   1. *Vaccination-related events or activities in the community such as health talks, compound meetings, etc.* 2. *Intervention design such as involvement in focus group discussions, surveys, interviews to pilot an intervention design* 3. *Programme delivery as a volunteer, peer support provider or other* 4. *Research such as participation in a study, trial or evaluation* 5. *Health care policy making such as decision making as it relates to health* 6. *Any stages of a community-directed coalition or programme* 7. *Advisory group of a community coalition or programme* 8. *Community health programme management*   *Provide example for any activity involved* |

**Evaluating SMS messaging for immunization demand generation in Nigeria: An Immunization Reminder and Information SMS System (IRISS) Project**

**JHU IRB No:** IRB00008732

**NHREC:** NHREC/01/01/2007-29/08/2019B

**Key Informant Interview for RI partners at State level.**

The interview will be held with RI partners at State level. (WHO, Unicef).

|  | **Introduction** | According to NDHS 2018, RI coverage and demand for vaccines in Kebbi State is very poor at 11%. As much as 21% of children had never received any vaccine from the RI program, and only 6% were fully vaccinated by two years of age. Poor demand for vaccination has been attributed to lack of information and knowledge about the vaccination program. The IRISS project aims to overcome the information gap with SMS messaging and reminders to caregivers to increase vaccine demand and uptake and improve public confidence in the immunization system. |
| --- | --- | --- |
|  | **Purpose of this interview**: | This interview guide aims to:   1. Understand changes in the RI program context in the State in the last one year 2. Evaluate availability of RI services in the State in the last one year. 3. Assess State’s readiness to scale up the intervention. 4. Assess programmatic feasibility of delivering the IRISS intervention. 5. Understand barriers to immunization uptake and social norms about vaccination in the last one year. |
|  | **Guidance to the interviewer**: | *Before the interview:*   1. Before beginning the interview, read the consent to the potential participant. 2. Encourage the potential participant to ask any clarifying questions and query him/her for understanding. 3. Ask for an oral permission to interview the participant.   *During the interview:*   1. Note: Each topic corresponds with a set of questions. 2. These questions are open-ended in nature.   *End of the interview:*   1. Thank the participants for their time. 2. Answer any remaining questions. 3. Provide the contact information if they have any questions or concerns:  - Dr. Chizoba Wonodi, Principal Investigator   Email: [cwonodi1@jhsph.edu](mailto:cwonodi1@jhsph.edu) or chizobabw@gmail.com   - Chisom Obi-Jeff, Project Coordinator   Email: [chisom.obi@dclnigeria.com](mailto:chisom.obi@dclnigeria.com) |

**RI program context**

| **S/N** | **Main question** | **Probes** |
| --- | --- | --- |
| 1. | In your view, what has been the most significant change in the RI program in the last one year? Provide examples | Any reasons? |
| 1.  2. | In the last one year, what new immunization programs were introduced in your State by national, state, partners and CSOs? | 1. *What are the activities?* 2. *How many LGAs are covered?* 3. *What is the duration of the program?* 4. *What successes have been recorded with the program?* 5. *What challenges have been recorded with the program?* 6. *What are the lessons learnt?* 7. *Who is funding it?* 8. *What other special programs are being conducted in your State? Describe* |
| 3 3. | In the last one year, what new RI partners work in the State? | 1. *Which program do each partner support?* 2. *Which aspects of immunization programs do each partner support?* 3. *What RI services do they provide?* 4. *Are all the LGAs covered?* 5. *Any report or material to provide more details?* |
| 2. 4.  2. | In the last one year, were there any program that involve working with community leaders (e.g. traditional/religious leaders), health workers, Ward CEFPs etc. in the State? | 1. *If yes, please list them* 2. *Are they active?* 3. *What activities do they conduct?* |
| 5. | Do you have specific program where community sensitization and reminder and education on vaccination is conducted in your State in the last one year? | 1. *What is the name of the program?* 2. *Who organizes such program?* 3. *What does the program entail?* |

**Availability of RI services**

| **S/N** | **Main question** | **Probes** |
| --- | --- | --- |
| 6. | According to Q3 2019 LQAS, RI coverage in Kebbi State is low. What could be the reason? | 1. *Any knowledge on the most common reasons mothers do not vaccinate their children on time and completely in your State?* 2. *As a State program manager, are there other reasons you think that lead to low RI coverage in your State?* |
| 7. | What has disrupted RI services in your State in the last one year, if any? Provide examples | 1. *Any vaccine stock outs and other RI commodities e.g AD syringes* 2. *Do health workers conduct their fixed and outreach sessions as stated in their microplans, If not why?* |
| 8. | What are the major challenges in delivering RI services in your State in the last one year, if any? | 1. ***Supply side challenges:*** 2. *Funding for RI activities,* 3. *Availability of cold chain equipment and health workers to conduct the RI sessions,* 4. *Health systems governance: How is it managed at the state, LGA and HF level? If the issue is to be fixed, what will it take? Who do we need to fix the issue?* 5. ***Demand side challenges:*** 6. *Community opposition to vaccination,* 7. *Immunization services not getting to community members* 8. *Lack of knowledge or empowerment to demand RI services from community members* 9. *Poor community engagement* |
| 9. | How best can RI services and coverage in your State be improved? Provide examples | *What resources are needed?* |

**IRISS implementation**

IRISS also known as Tunatar da ni is an intervention that use SMS to inform and educate caregivers/parents about the importance of immunization; and remind them of their child’s immunization schedules, including health facility vaccination schedules and service availability in their locality. IRISS deliver these immunization messages in three ways:

1. **General broadcasts** to the public with informative messages about immunization in general and advertisements for parents to opt-in to IRISS e-registry.
2. **Targeted broadcasts** to the community gatekeepers (District Heads, Village Heads, Imams) with messages on the importance of immunization and immunization clinic days of the health centers within the ward that offer RI.
3. **Individualized reminders** of child’s vaccination schedule to caregivers who register into IRISS e-registry

| **S/N** | **Main question** | **Probes** |
| --- | --- | --- |
| 10. | Have you heard of Tunatar dani? | 1. *If yes, how did you hear about Tunatar da ni?* 2. *What is Tunatar da ni about?* 3. *Have you ever received any text messages from Tunatar da ni? If yes, what is the message about?* 4. *Who in your State have you heard that receives messages from Tunatar dani if any?* |
| 11. | What are your views about Tunatar da ni on vaccination programs post-implementation? | 1. *Was it needed and why was it needed?* 2. *Any concern and how was it be addressed?* 3. *If people don’t have access to a mobile phone, how best can we send message to them?* 4. *What will make people pay attention or open a text message sent to them?* 5. *What aspects/components do you think should be or should have been included in the Tunatar da ni intervention?* 6. *What do you think should be or should have been included in the SMS reminders that would be vital for (i) caregivers, (ii)health workers (iii) program managers* |
| 12. | In the course of the project, majority of the registrations were done by RI providers who use their phone to register caregivers in order to receive the reminder on their phones. What do you feel about this on the impact of the RI program? | 1. *Does this change your level of interest for scale up, if any?* 2. *What are your thoughts about using Tunatar da ni for defaulter tracking? Please describe* |
| 13. | As a Partner, what are the efforts you made to ensure RI providers register caregivers for Tunatar da ni to receive personalized information about their child’s vaccination due dates? | 1. *Any reason for that?* 2. *Any reason for not willing to provide the support?* 3. *What will or did motivate mothers in the community to register for Tunatar da ni?* |
| 14. | One year ago, we trained community leaders (i.e. District Heads, Village Heads and Mai-ungwas), health workers and LGA program managers (including community engagement focal persons at LGA and Ward levels) to help mothers that cannot read and write register for Tunatar da ni to receive personalized information about their child’s vaccination due dates. What is your view about this? | 1. *What in your knowledge motivated them to provide this service to parents?* 2. *What barriers did they face in providing this service, and how was it be mitigated? Provide examples* 3. *Is there another approach we would have used to help mothers register for Tunatar da ni?* |

**Decision-making process**

| **S/N** | **Main question** | **Probes** |
| --- | --- | --- |
| 15. | At baseline, respondents told us that to adopt an intervention like IRISS, we need to present data on its effectiveness in improving RI coverage and its cost effectiveness.  Having implemented IRISS intervention for one year, is there any change in the way the state will make decisions to scale up or adopt *Tunatar da ni*? | 1. *What does it now require?* 2. *What new data/information is needed?* 3. *Any change in how long it will take?* 4. *Who will be the stakeholders involved?* |
| 16. | Assuming *Tunatar da ni* is considered for scale up, what role will state program managers play in the decision making process? | 1. *What is the role of different influencers: national, partners or other level of influencers?* 2. *Is it what the state can take up on their own or have to wait for approval from NPHCDA?* |
| 17. | How will Tunatar da ni sit in the state immunization program? | 1. *Which department will it be?* 2. *Who will manage it?* 3. *What other resources will you need?* |
| 18. | At baseline, some respondents mentioned that we should include Robo call as part of IRISS. To pass the same message, a one-minute Robo call costs X while a text message cost N2.50. Given this information, which will you choose for government to scale up? | 1. *What are your reasons?* |

**Barriers to immunization uptake and social norms about vaccination**

| **S/N** | **Main question** | **Probes** |
| --- | --- | --- |
| 19. | In the last one year, how did community leaders (religious or political or traditional leaders), health workers, program managers at State and LGA support vaccination for infants and children in your state/community? Provide examples on the type of support | 1. *What specific role did they play? Provide examples* 2. *What about support in advocating or encouraging registration for Tunatar da ni* *in the last one year? Provide examples* 3. *What about support in accessing vaccination in the last one year? Provide example* 4. *What about supporting community engagement in the last one year? Provide example* 5. *In the last one year, has there been any pressure to vaccinate children in the community, and from who? Provide examples* |
| 20 | In the last one year, how did you promote immunization in your State? Provide examples | 1. *What specific role did you play?* 2. *What about support in accessing vaccination?* 3. *What about supporting community engagement in the last one year? Provide example* 4. *Any pressure to vaccinate children in the community, and from who? Provide examples* |
| 21 | In the last one year, what influenced your decision to promote immunizations in the State? | 1. *Provide examples* 2. *Any pressure from anyone?* |

**Evaluating SMS messaging for immunization demand generation in Nigeria: An Immunization Reminder and Information SMS System (IRISS) Project**

**JHU IRB No:** IRB00008732

**NHREC:** NHREC/01/01/2007-29/08/2019B

**Key Information Interview Guide for Policymakers (State Primary Health Care Development Agency Leadership)**

The interview will be held with the Executive Secretary Kebbi State Primary Health Care Development Agency

|  | **Introduction** | According to NDHS 2018, RI coverage and demand for vaccines in Kebbi State is very poor at 11%. As much as 21% of children had never received any vaccine from the RI program, and only 6% were fully vaccinated by two years of age. Poor demand for vaccination has been attributed to lack of information and knowledge about the vaccination program. The IRISS project aims to overcome the information gap with SMS messaging and reminders to caregivers to increase vaccine demand and uptake and improve public confidence in the immunization system. |
| --- | --- | --- |
|  | **Purpose of this interview**: | This interview guide aims to:   1. Assess change in political priority for routine immunization (RI) in the State in the last one year 2. Understand the decision-making process around scale up of new interventions such as IRISS 3. Understand policy makers views on the acceptability and challenges of implementing the intervention 4. Understand change in barriers to immunization uptake and social norms about vaccination in the last one year. |
|  | **Guidance to the interviewer**: | *Before the interview:*   1. Before beginning the interview, read the consent to the potential participant. 2. Encourage the potential participant to ask any clarifying questions and query him/her for understanding. 3. Ask for an oral permission to interview the participant.   *During the interview:*   1. Note: Each topic corresponds with a set of questions. 2. These questions are open-ended in nature.   *End of the interview:*   1. Thank the participants for their time. 2. Answer any remaining questions. 3. Provide the contact information if they have any questions or concerns:  - Dr. Chizoba Wonodi, Principal Investigator   Email: [cwonodi1@jhsph.edu](mailto:cwonodi1@jhsph.edu) or chizobabw@gmail.com   - Chisom Obi-Jeff, Project Coordinator   Email: [chisom.obi@dclnigeria.com](mailto:chisom.obi@dclnigeria.com) |

**Prioritization of RI in the State**

| **S/N** | **Main question** | **Probes** |
| --- | --- | --- |
| 1.  1. | At baseline, you mentioned RI among the three health priorities in the State. Is it still the same in the last one year? | 1. *If yes, why? Provide examples* 2. *If no, why? Provide examples* |
| 2. | According to the NDHS 2018, RI coverage in Kebbi State is 11%. What are the major causes of this low immunization coverage in your state in the last one year? | *Probe for reasons:*   1. *Is it* ***supply side*** *(funding, unavailability of health workers to conduct RI session, health infrastructure, vaccine stock out, governance etc.) or* 2. *Is it* ***demand side*** *(community opposition to vaccination, rumors about vaccination, fear about vaccine side effects, people are not coming for immunization or immunization services are not getting to them, lack of knowledge or empowerment to demand RI services from community members) challenges* |
| 3. | What about the most common reasons mothers do not vaccinate their children on time and completely in your State in the last one year? | *What is the best way to motivate caregivers/parents to take their children for vaccination on time and completely?* |
| 4. | In the last one year, how do you support vaccination for infants and children in your State? Provide examples | 1. *What specific role did you play?* 2. *What about support in accessing vaccination?* 3. *Any pressure to vaccinate children in your state, and from who? Provide examples* |
| 5. | At baseline, you mentioned that you’d prioritize interventions to fix the demand side challenge. Will you still prioritize interventions to fix the demand side challenges? If yes, how | 1. *What are the reasons for the choice you made?* 2. *What are the potential challenges and how do you plan to mitigate them?* |

**IRISS implementation**

IRISS also known as Tunatar da ni is an intervention that use SMS to inform and educate caregivers/parents about the importance of immunization; and remind them of their child’s immunization schedules, including health facility vaccination schedules and service availability in their locality. IRISS delivered these immunization messages in three ways:

1. **General broadcasts** to the public with informative messages about immunization in general and advertisements for parents to opt-in to IRISS e-registry.
2. **Targeted broadcasts** to the community gatekeepers (District Heads, Village Heads, Imams) with messages on the importance of immunization and immunization clinic days of the health centers within the ward that offer RI.
3. **Individualized reminders** of child’s vaccination schedule to caregivers who register into IRISS e-registry

| **S/N** | **Main question** | **Probes** |
| --- | --- | --- |
| 6. | Have you heard of Tunatar dani? | 1. *If yes, how did you hear about Tunatar da ni?* 2. *What is Tunatar da ni about?* 3. *Have you ever received any text messages from Tunatar da ni? If yes, what is the message about?* 4. *Who in your State have you heard that receives messages from Tunatar dani if any?* |
| 7. | What are your views about Tunatar da ni on vaccination programs post-implementation? | 1. *Was it needed and why was it needed?* 2. *Any concern and how was or can it be addressed?* 3. *If people don’t have access to a mobile phone, how best can we send message to them?* 4. *What will make people pay attention or open a text message sent to them?* 5. *What aspects/components do you think should be or should have been included in the Tunatar da ni intervention?* 6. *What do you think should be or should have been included in the SMS reminders that would be vital for (i) caregivers, (ii)health workers (iii) program managers* |
| 8. | How did you advocate for its adoption in your State? Provide examples | 1. *Any reason for that?* 2. *Any reason for not advocating?* |
| 9. | One year ago, we trained community leaders (i.e. District Heads, Village Heads and Mai-ungwas), health workers and LGA program managers (including community engagement focal persons at LGA and Ward levels) to help mothers that cannot read and write register for Tunatar da ni to receive personalized information about their child’s vaccination due dates. What is your view about this? | 1. *What in your knowledge motivated them to provide this service to parents?* 2. *What barriers did they face in providing this service, and how was it be mitigated?* 3. *Any political opposition to using these people for this kind of intervention?* 4. *Is there another approach we would have used to help mothers register for Tunatar dani?* 5. *What in your opinion will motivate mothers in the community to register for Tunatar da ni?* |

**Decision-making process**

| **S/N** | **Main question** | **Probes** |
| --- | --- | --- |
| 10. | At baseline, respondents told us that to adopt an intervention like IRISS, we need to present data on its effectiveness in improving RI coverage and its cost effectiveness.  Having implemented IRISS intervention for one year, is there any change in the way the state will make decisions to scale up or adopt *Tunatar da ni*? | 1. *What does it now require?* 2. *What new data/information is needed?* 3. *Any change in how long it will take?* 4. *Who will be the stakeholders involved?* |
| 11. | Assuming *Tunatar da ni* is considered for scale up, what role will you play in the decision-making process? | 1. *What is the role of different influencers: e.g. national, partners or other level of influencers?* 2. *Is it what the state can take up on their own or have to wait for approval from NPHCDA?* |
| 12. | How will Tunatar da nisit in the state immunization program? | 1. *Which department will it be?* 2. *Who will manage it?* 3. *What other resources will you need?* |
| 13. | At baseline, some respondents mentioned that we should include Robo call as part of IRISS. To pass the same message, a one-minute Robo call costs X while a text message cost N2.50. Given this information, which will you choose for government to scale up? | 1. *What are your reasons?* |

**Barriers to immunization uptake and social norms about vaccination**

| **S/N** | **Main question** | **Probes** |
| --- | --- | --- |
| 14. | In the last one year, how do program managers at State and LGA support vaccination for infants and children in your state/community? Provide examples on the type of support | 1. *What specific role did they play? Provide examples* 2. *What about support in advocating or encouraging registration for Tunatar da ni* *in the last one year? Provide examples* 3. *What about support in accessing vaccination in the last one year? Provide example?* 4. *In the last one year, has there been any pressure to vaccinate children in the community, and from who? Provide examples* |
| 15. | In the last one year, what influenced your decision to promote immunizations in your State? | 1. *Provide examples* |

**Evaluating SMS messaging for immunization demand generation in Nigeria: An Immunization Reminder and Information SMS System (IRISS) Project**

**JHU IRB No:** IRB00008732

**NHREC:** NHREC/01/01/2007-29/08/2019B

**Key Informant Interview for State immunization program managers.**

The interview will be held with the State Director of Immunization Officer and PM SERICC

|  | **Introduction** | According to NDHS 2018, RI coverage and demand for vaccines in Kebbi State is very poor at 11%. As much as 21% of children had never received any vaccine from the RI program, and only 6% were fully vaccinated by two years of age. Poor demand for vaccination has been attributed to lack of information and knowledge about the vaccination program. The IRISS project aims to overcome the information gap with SMS messaging and reminders to caregivers to increase vaccine demand and uptake and improve public confidence in the immunization system. |
| --- | --- | --- |
|  | **Purpose of this interview**: | This interview guide aims to:   1. Understand changes in the RI program context in the last one year 2. Evaluate availability of RI services in the last one year 3. Assess State’s readiness to scale up the intervention. 4. Assess programmatic feasibility of delivering the IRISS intervention. 5. Understand barriers to immunization uptake and social norms about vaccination in the last one year. |
|  | **Guidance to the interviewer**: | *Before the interview:*   1. Before beginning the interview, read the consent to the potential participant. 2. Encourage the potential participant to ask any clarifying questions and query him/her for understanding. 3. Ask for an oral permission to interview the participant.   *During the interview:*   1. Note: Each topic corresponds with a set of questions. 2. These questions are open-ended in nature.   *End of the interview:*   1. Thank the participants for their time. 2. Answer any remaining questions. 3. Provide the contact information if they have any questions or concerns:  - Dr. Chizoba Wonodi, Principal Investigator   Email: [cwonodi1@jhsph.edu](mailto:cwonodi1@jhsph.edu) or chizobabw@gmail.com   - Chisom Obi-Jeff, Project Coordinator   Email: [chisom.obi@dclnigeria.com](mailto:chisom.obi@dclnigeria.com) |

**RI program context**

| **S/N** | **Main question** | **Probes** |
| --- | --- | --- |
| 1. | In your view, what has been the most significant change in the RI program in the last one year? Provide examples | Any reasons? |
| 1.  2. | In the last one year, what new immunization programs were introduced in your State by national, state, partners and CSOs? | 1. *What are the activities?* 2. *How many LGAs are covered?* 3. *What is the duration of the program?* 4. *What successes have been recorded with the program?* 5. *What challenges have been recorded with the program?* 6. *What are the lessons learnt?* 7. *Who is funding it?* 8. *What other special programs are being conducted in your State? Describe* |
| 3 3. | In the last one year, what new RI partners work in the State? | 1. *Which program do each partner support?* 2. *Which aspects of immunization programs do each partner support?* 3. *What RI services do they provide?* 4. *Are all the LGAs covered?* 5. *Any report or material to provide more details?* |
| 2. 4.  2. | In the last one year, were there any RI program that involved working with community leaders (eg. traditional/religious leaders), health workers, Ward CEFPs etc. in the State? | 1. *If yes, please list them* 2. *Are they active?* 3. *What activities do they conduct?* |
| 5. | Do you have specific program where community sensitization and reminder and education on vaccination is conducted in your State in the last one year? | 1. *What is the name of the program?* 2. *Who organizes such program?* 3. *What does the program entail?* |

**Availability of RI services**

| **S/N** | **Main question** | **Probes** |
| --- | --- | --- |
| 6. | According to Q3 2019 LQAS, RI coverage in Kebbi State is low. What could be the reason? | 1. *Any knowledge on the most common reasons mothers do not vaccinate their children on time and completely in your State?* 2. *As a State program manager, are there other reasons you think that lead to low RI coverage in your State?* |
| 7. | What has disrupted RI services in your State in the last one year, if any? Provide examples | 1. *Any vaccine stock outs and other RI commodities e.g AD syringes* 2. *Do health workers conduct their fixed and outreach sessions as stated in their microplans, If not why?* |
| 8. | What are the major challenges in delivering RI services in your State in the last one year, if any? | 1. ***Supply side challenges:*** 2. *Funding for RI activities,* 3. *Availability of cold chain equipment and health workers to conduct the RI sessions,* 4. *Health systems governance: How is it managed at the state, LGA and HF level? If the issue is to be fixed, what will it take? Who do we need to fix the issue?* 5. ***Demand side challenges:*** 6. *Community opposition to vaccination,* 7. *Immunization services not getting to community members* 8. *Lack of knowledge or empowerment to demand RI services from community members* 9. *Poor community engagement* |
| 9. | How best can RI services and coverage in your State be improved? Provide examples | *a. What resources are needed?* |

**IRISS implementation**

IRISS also known as Tunatar da ni is an intervention that use SMS to inform and educate caregivers/parents about the importance of immunization; and remind them of their child’s immunization schedules, including health facility vaccination schedules and service availability in their locality. IRISS deliver these immunization messages in 3 ways:

1. **General broadcasts** to the public with informative messages about immunization in general and advertisements for parents to opt-in to IRISS e-registry.
2. **Targeted broadcasts** to the community gatekeepers (District Heads, Village Heads, Imams) with messages on the importance of immunization and immunization clinic days of the health centers within the ward that offer RI.
3. **Individualized reminders** of child’s vaccination schedule to caregivers who register into IRISS e-registry

| **S/N** | **Main question** | **Probes** |
| --- | --- | --- |
| 10. | Have you heard of Tunatar dani? | 1. *If yes, how did you hear about Tunatar da ni?* 2. *What is Tunatar da ni about?* 3. *Have you ever received any text messages from Tunatar da ni? If yes, what is the message about?* 4. *Who in your State have you heard that receives messages from Tunatar dani if any?* |
| 11. | What are your views about Tunatar da ni on vaccination programs post-implementation? | 1. *Was it needed and why was it needed?* 2. *Any concern and how was or can it be addressed?* 3. *If people don’t have access to a mobile phone, how best can we send message to them?* 4. *What will make people pay attention or open a text message sent to them?* 5. *What aspects/components do you think should be or should have been included in the Tunatar da ni intervention?* 6. *What do you think should be or should have been included in the SMS reminders that would be vital for (i) caregivers, (ii)health workers (iii) program managers* |
| 12. | In the course of the project, majority of the registrations were done by RI providers who use their phone to register caregivers in order to receive the reminder on their phones. What do you feel about this on the impact of the RI program? | 1. *Does this change your level of interest for scale up, if any?* 2. *What are your thoughts about using Tunatar da ni for defaulter tracking? Please describe* |
| 13. | As a program manager, what are the efforts you made to ensure RI providers register caregivers for Tunatar da ni to receive personalized information about their child’s vaccination due dates? | 1. *Any reason for that?* 2. *Any reason for not willing to provide the support?* 3. *What will or did motivate RI providers in the community to register caregivers for Tunatar da ni?* |
| 14. | One year ago, we trained community leaders (i.e. District Heads, Village Heads and Mai-ungwas), health workers and LGA program managers (including community engagement focal persons at LGA and Ward levels) to help mothers that cannot read and write register for Tunatar da ni to receive personalized information about their child’s vaccination due dates. What is your view about this? | 1. *What in your knowledge motivated them to provide this service to parents?* 2. *What barriers did they face in providing this service, and how was it mitigated? Provide examples* 3. *Is there another approach we would have used to help mothers register for Tunatar da ni if any?* |

**Decision-making process**

| **S/N** | **Main question** | **Probes** |
| --- | --- | --- |
| 15. | At baseline, respondents told us that to adopt an intervention like IRISS, we need to present data on its effectiveness in improving RI coverage and its cost effectiveness.  Having implemented IRISS intervention for one year, is there any change in the way the state will make decisions to scale up or adopt *Tunatar da ni*? | 1. *What does it now require?* 2. *What new data/information is needed?* 3. *Any change in how long it will take?* 4. *Who will be the stakeholders involved?* |
| 16. | Assuming *Tunatar da ni* is considered for scale up, what role will state program managers play in the decision making process? | 1. *What is the role of different influencers: national, partners or other level of influencers?* 2. *Is it what the state can take up on their own or have to wait for approval from NPHCDA?* |
| 17. | How will Tunatar da ni sit in the state immunization program? | 1. *Which department will it be?* 2. *Who will manage it?* 3. *What other resources will you need?* |
| 18. | At baseline, some respondents mentioned that we should include Robo call as part of IRISS. To pass the same message, a one-minute Robo call costs X while a text message cost N2.50. Given this information, which will you choose for government to scale up? | *What are your reasons?* |

**Barriers to immunization uptake and social norms about vaccination**

| **S/N** | **Main question** | **Probes** |
| --- | --- | --- |
| 19. | In the last one year, how did community leaders (religious or political or traditional leaders), health workers, program managers at LGA support vaccination for infants and children in your state? Provide examples on the type of support | 1. *What specific role did they play? Provide examples* 2. *What about support in advocating or encouraging registration for Tunatar da ni* *in the last one year? Provide examples* 3. *What about support in accessing vaccination in the last one year? Provide example* 4. *What about supporting community engagement in the last one year? Provide example* 5. *In the last one year, has there been any pressure to vaccinate children in the State, and from who? Provide examples* |
| 20 | In the last one year, how did you promote immunization in your State? Provide examples | 1. *What specific role did you play?* 2. *What about support in accessing vaccination?* 3. *What about supporting community engagement in the last one year? Provide example* 4. *Any pressure to vaccinate children in the community, and from who? Provide examples* |
| 21. | In the last one year, what influenced your decision to promote immunizations in your state? | *Provide examples* |
