# Supplementary material for: Implementing SMS reminders for routine immunization in Northern Nigeria: a qualitative evaluation using the RE-AIM framework
Source: BMC Public Health. 2022 Dec 17;22:2370. doi: 10.1186/s12889-022-14822-1 (PMC9758467; doi:10.1186/s12889-022-14822-1)
Supplement: Supplementary file 3 — Additional file 3. Complete list of participants interviewed and type of interviewconducted. [file 12889_2022_14822_MOESM3_ESM.docx]

**Additional File 3.** Full list of participants interviewed, and type of interview conducted

| **S/N** | **Type of respondent** | **No. and type of interview** | **No. of respondents** |
| --- | --- | --- | --- |
|  | State leadership | 1 KII | 1 |
|  | State-level routine immunization program managers | 2 KIIs | 2 |
|  | Routine Immunization Partners at State level | 2 KIIs | 2 |
|  | Routine Immunization providers at the facility level, including ward focal person | 10 KIIs | 10 |
|  | LGA routine immunization program managers | 5 KIIs | 5 |
|  | Fathers of children under 5 years of age | 3 FGDs | 23 |
|  | Mothers of children under 5 years of age | 3 FGDs | 24 |
|  | Male youth | 1 FGD | 8 |
|  | Female youth | 1 FGD | 8 |
|  | Traditional Birth Attendants | 1 FGD | 8 |
|  | WDC members | 2 FGDs | 16 |
|  | Opinion leaders (traditional and religious leaders, persons of influence) | 3 FGDs | 24 |
|  | Parents of children under 5 years | 2 IDIs | 2 |
|  | Young men | 2 IDIs | 2 |
|  | Young women | 2 IDIs | 2 |
|  | Opinion leaders | 4 IDIs | 4 |
|  | Parents of newborn | 2 IDIs | 2 |
|  | Pregnant women | 1 IDI | 1 |
| **TOTAL** | | | **144** |
